# Multiple Distinct Stimuli Increase Measured Nucleosome Occupancy around Human Promoters

**DOI:** 10.1371/journal.pone.0023490

**Published:** 2011-08-11

**Authors:** Chuong D. Pham, Hillel I. Sims, Trevor K. Archer, Gavin R. Schnitzler

**Affiliations:** 1 AstraZeneca R&D Boston, Waltham, Massachusetts, United States of America; 2 Department of Biology, Rosenstiel Basic Medical Sciences Research Center, Brandeis University, Waltham, Massachusetts, United States of America; 3 Laboratory of Molecular Carcinogenesis, National Institute of Environmental Health Sciences, National Institutes of Health, Research Triangle Park, North Carolina, United States of America; 4 Molecular Cardiology Research Institute, Tufts Medical Center, Boston, Massachusetts, United States of America; Baylor College of Medicine, United States of America

## Abstract

Nucleosomes can block access to transcription factors. Thus the precise localization of nucleosomes relative to transcription start sites and other factor binding sites is expected to be a critical component of transcriptional regulation. Recently developed microarray approaches have allowed the rapid mapping of nucleosome positions over hundreds of kilobases (kb) of human genomic DNA, although these approaches have not yet been widely used to measure chromatin changes associated with changes in transcription. Here, we use custom tiling microarrays to reveal changes in nucleosome positions and abundance that occur when hormone-bound glucocorticoid receptor (GR) binds to sites near target gene promoters in human osteosarcoma cells. The most striking change is an increase in measured nucleosome occupancy at sites spanning ∼1 kb upstream and downstream of transcription start sites, which occurs one hour after addition of hormone, but is lost at 4 hours. Unexpectedly, this increase was seen both on GR-regulated and GR-non-regulated genes. In addition, the human SWI/SNF chromatin remodeling factor (a GR co-activator) was found to be important for increased occupancy upon hormone treatment and also for low nucleosome occupancy without hormone. Most surprisingly, similar increases in nucleosome occupancy were also seen on both regulated and non-regulated promoters during differentiation of human myeloid leukemia cells and upon activation of human CD4+ T-cells. These results indicate that dramatic changes in chromatin structure over ∼2 kb of human promoters may occur genomewide and in response to a variety of stimuli, and suggest novel models for transcriptional regulation.

## Introduction

Nucleosomes can inhibit transcription by blocking the access of transcription factors to their sites on DNA. Since the linker DNA between nucleosomes (which averages ∼60 bp in length) is far more accessible than nucleosome bound DNA, the precise location of nucleosomes on DNA will functionally control transcription factor binding. Recent studies have established genomic microarray and multiparallel sequencing approaches for mapping endogenous nucleosome positions that can be orders of magnitude more efficient than standard approaches, such as indirect end labeling [1], [2], [3], [4], [5], [6], [7]. Strikingly, these studies have shown that ∼80% of all yeast nucleosomes adopt specific positions relative to the underlying DNA sequence [1], [2], [3], [4], [8], [9], [10], [11], and have indicated that positioned nucleosomes are also common in a variety of organisms from *C. Elegans* to man [5], [6], [7], [10], [12]. This sequence-directed arrangement of nucleosomes is likely to directly impact transcription, since functional transcription factor binding sites (consensus sites that are evolutionarily conserved and/or known to be bound *in vivo*) were much more frequently found in linker regions than in DNA covered by nucleosomes, and since start sites of active genes were frequently devoid of nucleosomes [2], [3], [4], [6], [13]. These genomic mapping approaches can also be used to relate changes in nucleosome positions with changes in gene expression. For instance, two recent studies in yeast showed that heat shock gene activation was frequently associated with decreased nucleosome occupancy over start sites, while repression was associated with increased occupancy, effects which were often dependent on yeast SWI/SNF function and correlated with SWI/SNF binding [11], [14]. These studies indicate that nucleosome positioning will be involved in transcriptional regulation much more often than was initially suspected, and emphasize the need for a deeper understanding of how nucleosome positions are functionally controlled.

Glucocorticoid agonists are some of the most commonly prescribed drugs to treat inflammation and a variety of immune disorders [15], [16]. Binding of cortisol, dexamethasone or other glucocorticoid agonists causes a conformational change in the Glucocorticoid Receptor (GR), releasing it from cytoplasmic heat shock proteins, allowing it to dimerize, translocate into the nucleus and bind to glucocorticoid response elements (GREs) at target gene loci. Hormone binding also facilitates interaction with coactivator complexes including the ATP-dependent chromatin remodeling complex, SWI/SNF (which is a required co-activator for GR as well as many other nuclear hormone receptors [17]). Recent studies have identified a growing number of genes that are directly activated or repressed by binding of dexamethasone-bound GR [18], [19], [20], [21], [22], [23], [24], [25], [26]. In most cases, however, little is known about the chromatin structure of these genes' promoters or remodeling events that accompany GR binding. Some clues do exist, however. For instance, introduction of functional human SWI/SNF (hSWI/SNF) into cells that lack it greatly increased the accessibility of DNA normally covered by one Mouse Mammary Tumor Virus (*MMTV*) promoter nucleosome, Nuc B, which occupies the promoter from ∼−250 to −100 and covers GRE elements as well as an essential NF1 binding site ([19], [27], [28], [29], [30], [31], and for review see [32]). In addition, one recent study examined DNase hypersensitive sites on ten mouse genes that were either activated or repressed by GR, most of which were also regulated by SWI/SNF [24]. The results showed that GR- and/or hSWI/SNF-dependent increases in DNase sensitivity were found at many of these loci, frequently mapping near GR binding sites. These and other studies indicate that chromatin changes, driven at least in part by hSWI/SNF, are important aspects of gene regulation by GR. However, the specific nature of these changes is largely unknown.

Here, we make use of genomic tiling microarray technology to examine chromatin changes on GR-regulated genes when human cells are treated with a dexamethasone, using siRNA-mediated knock down of the BRG1 ATPase to examine the role of hSWI/SNF in mediating these changes. We also examine changes in promoter chromatin that occur during T cell activation [5], and during differentiation of human myeloid leukemia cells into granulocytes. At individual promoters, we identified some discrete changes in nucleosome occupancy that occur in response to dexamethasone and/or as a result of BRG1 knock down. Most strikingly, however, we found that the diverse inducing signals in all three systems resulted in large apparent increases in nucleosome occupancy over ∼2 kb surrounding Pol II transcription start sites, both for genes that are regulated by these stimuli as well as genes that are not. These unexpected findings suggest that genomewide alterations in promoter nucleosome abundance may occur in response to a variety of stimuli, and give rise to novel models for gene regulation in chromatin.

## Results

We designed a custom NimbleGen human genomic microarray containing 50 mer oligonucleotides offset from each other by 10 bp (e.g. each oligo overlaps its nearest neighbor by 40 bp), as per [6], [12]. The array contained the entire transcribed region, 20 kb of 5′ untranscribed DNA and 7 kb of 3′ untranscribed DNA of about two dozen human genes (see tables S1 and S2). These include ten GR/dex activated and four GR/dex repressed genes. For most of these, the location of GR binding site(s) in the promoter or upstream untranscribed DNA has been mapped [18], [19], [20], [21], [22], [23]. The array also covered several genes that are not regulated by GR: including, *CSF1* and *CD44* (which are highly dependent on hSWI/SNF [33], [34], [35]), *CDK1*, *E2F1*, *CCNE1*, *CCNB2*, *CCNA1* (cell cycle control genes that are variously repressed or activated by hSWI/SNF complexes [36], [37], [38], [39], [40], [41]), and the housekeeping core metabolic gene *GAPDH* (whose expression is independent of GR and hSWI/SNF [42], [43], [44], [45]). To reduce high-frequency noise that was evident in early human nucleosome microarray mapping studies [6], [12], we tiled each sequence onto the array four times (two forward and two reverse oligos), an approach which was shown to be effective in one small-scale study [12].

**Figure 1:**
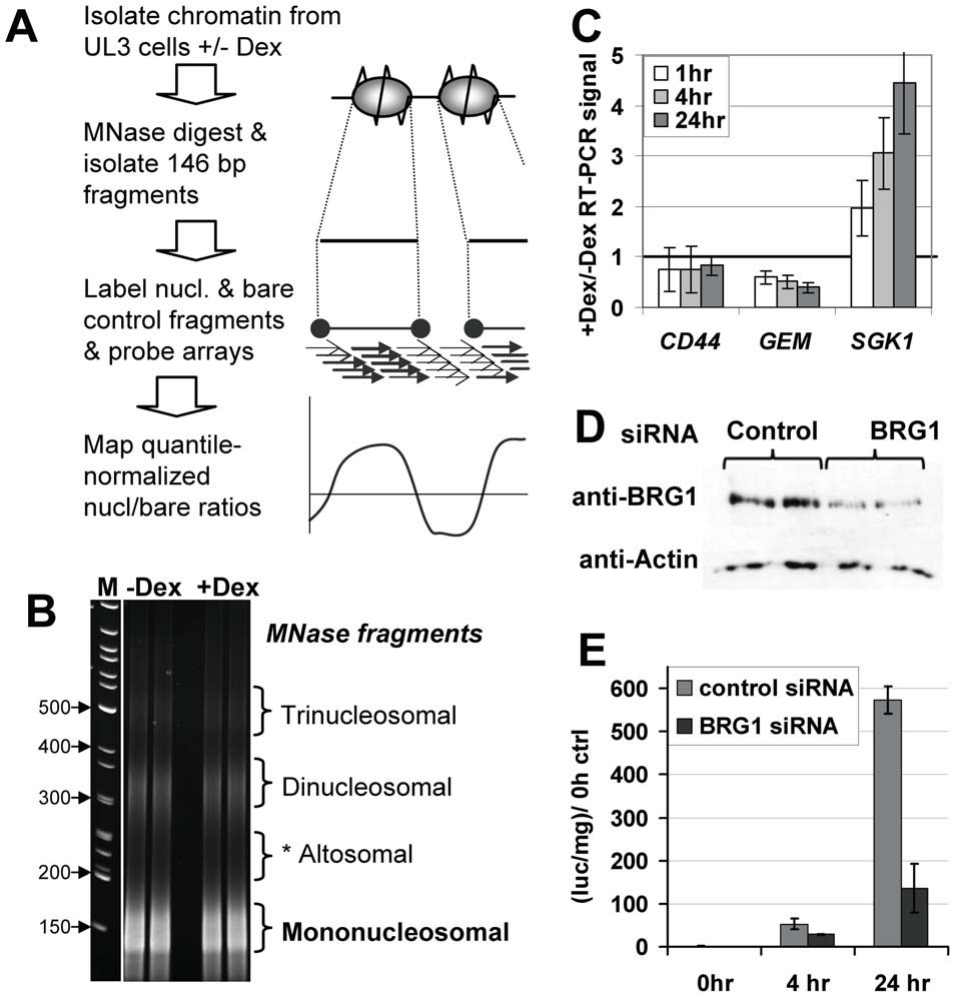
System for the analysis of chromatin changes during glucocorticoid treatment. (**A**) Experimental schematic. (**B**) Example of PAGE of chromatin fragments used to isolated mononucleosomal fragments (as well as altosomal fragments analyzed in Fig. S15). Complete gels and quantitation are shown in Fig. S1. (**C**) RT-PCR for mRNA levels of *CD44* (control), *GEM* (Dex repressed) and *SGK1* (Dex activated) endogenous genes. Results show the signal at the indicated time with Dex divided by the signal without Dex. (**D**) Western blot versus BRG1, using anti-actin as a control, shows transfection with BRG1 specific siRNA reduces BRG1 protein levels. (**E**) *MMTV* luciferase transactivation by Dex, as measured by luminometer, is attenuated in BRG1 siRNA transfected cells.

To examine chromatin changes associated with transcriptional regulation by GR, we used UL3 cells, derivatives of U2-OS human osteosarcoma cells, which stably express GR and also contain a single integrated copy of a *MMTV*-luciferase transgene [28], [46]. As illustrated in Figure 1A, UL3 cells were treated with the glucocorticoid agonist dexamethasone (Dex) for 1 and 4 hours, or with ethanol vehicle alone, followed by chromatin isolation, digestion with MNase and isolation of ∼146 bp mononucleosome DNA fragments (a sample gel is shown in Fig. 1B). These fragments were then used to probe the array, using MNase digested bare DNA fragments as controls for variability in hybridization efficiency from oligo to oligo on the array [6], [12]. The nucleosome/bare ratio for each of the four oligos representing each position was determined, and median values for each position were quantile normalized and denoised using a novel deviation-weighted smoothing function (see Materials and Methods). The effect of Dex treatment on GR-regulated promoters was confirmed by measuring luciferase expression from the integrated *MMTV*-luc construct (Fig. 1E, light grey bars), and by RT-PCR measurement of mRNA levels of endogenous genes (Fig. 1C shows the effect of Dex treatment on *SGK1* (GR-activated), *GEM* (GR-repressed) and *CD44* (GR-unregulated)).

### Nucleosome occupancy profiles generated from tiling array data

**Figure 2:**
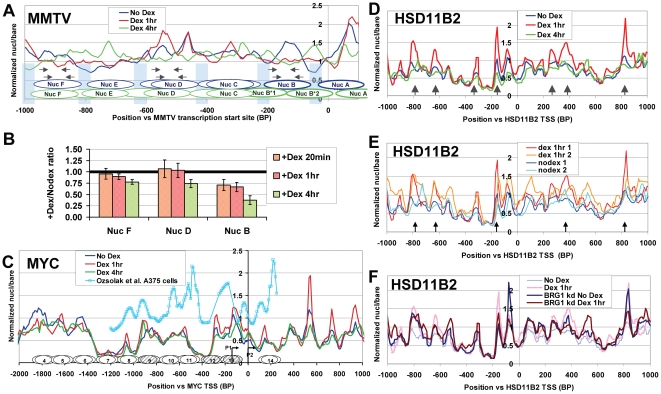
Hormone addition results in chromatin changes at individual GR-regulated genes. UL3 cells were transfected with BRG1-specific or control siRNA oligos, and treated, after 71 hours with 10 nM Dex (or ethanol vehicle) for 1 hr. Alternatively, untransfected cells were treated with Dex for 4 hrs. (**A**) Quantile normalized nucleosome/bare ratios for the *MMTV* LTR promoter region from the integrated MMTV-luciferase construct for No Dex (blue line), Dex 1 hr (red line) or Dex 4 hrs (green line). Blue bars: approximate locations of nucleosome boundaries from indirect end labeling experiments [19], [48]. Blue & green ovals: nucleosome positions for No Dex or Dex 4 hrs. Arrows: primer sets used to detect Nucs B, D & F for Fig. 2B. We calculate that a nucleosome/bare ratio of 1.0 corresponds to ∼50% nucleosome occupancy, while a value of 2.1 corresponds to 100% occupancy (e.g. a nucleosome covering a given location on all gene copies). Because a nucleosomal fragment must cover the full length of each oligo to give a strong signal, well positioned nucleosomes are represented by signal peaks greater than 1.0 and spanning only about 9 oligos (∼90 bp), an effect which also increases the apparent width of nucleosome-free linker regions. For more details, see Additional Methods, in Text S1. (**B**) MNase footprint PCR results for MMTV, using the primer sets indicated in (A). The PCR signal from mononucleosomal fragments at the indicated positions and times after Dex addition was normalized by dividing by the NoDex signal, and the results from both pairs of primers corresponding to each position averaged. Bars indicate standard error of the mean from 6 PCR reactions. In addition to the progressive decrease at Nuc D at 20 mins, 1 hr and especially 4 hrs, moderately decreased signal at Nucs D and F was also seen at 4 hrs (consistent, perhaps, with the somewhat lower occupancy, especially for Nuc D, seen in Fig. 2A). (**C**) As for (A), but showing the nucleosome profile for the Dex-repressed endogenous *MYC* promoter. Ovals indicate nucleosome positions mapped using indirect-end labeling in human HL60 cells [51], [52], [53]. The light blue line and squares shows array mapping results from A375 cells ([6], GEO # GSE6385). Arrows show the minor (P1) and major (P2) *MYC* promoters. (**D**) As for (A), but showing results for the Dex-induced endogenous *HSD11B2* promoter. Grey arrows indicate sites where nucleosome occupancy increases or decreases after 1 hr of dex treatment. (**E**) Comparison of No Dex and Dex 1 hr experimental results from two separate experiments. Conditions for the second experiment were identical, except that cells were not transfected with control siRNA oligos before hormone treatment. Arrows indicate sites of statistically significant nucleosome occupancy changes. We found that almost all locations that met this criterion for experiment #1 (e.g. dark blue and red curves at arrows) showed an occupancy difference in the same direction in experiment #2 (e.g. blue-green and orange curves at arrows). (**F**) No Dex and Dex 1 hr treatments in BRG1 knock down cells (dark blue and dark red lines). The data from control transfected cells (from (D)) is shown as stippled lines.

The nucleosome occupancy profiles for the *MMTV* and *MYC* promoters are presented in Fig. 2A and 2C. These genes are useful initial examples, because nucleosome positions on their promoters have been examined, in untreated cycling cells at least, using both indirect end-labeling and genomic tiling microarray approaches. In both cases, we found that our nucleosome coverage curves fit reasonably well with prior indirect end-labeling results ([12], [47], [48], [49], [50], [51], [52], [53], approximate nucleosome positions from which are indicated by blue ovals in Fig. 2A and black ovals in Fig. 2C). They also fit well with prior microarray mapping studies of *MMTV* in MDA-kb2 cells [12], and of c-myc in the human A375 melanoma line ([6], compare solid blue line to light blue squares in Fig. 2C). Note that these correspondences are not expected to be perfect because of differences in cell lines or conditions, and because of fundamental differences in the way end labeling and array hybridization detect nucleosome positions (as discussed further in Fig. S8A).

### Array mapping reveals novel gene-specific effects of Dex addition on GR-target genes

We found that Dex addition eliminated the positioned nucleosome over MMTV Nuc B after 1 hr. (Fig. 2A, compare red line to blue line). This is consistent with prior studies showing that DNA sequences in Nuc B become very sensitive to MNase and restriction enzymes after Dex treatment (e.g. [50], [54], [55]). We used the results of biological repeat –Dex and +Dex 1 hr samples to establish an estimated error for nucl/bare values for any given position, of +/−0.14. Using this value, we calculated that any case where the average nucl/bare ratio for a nucleosome sized peak (spanning ∼90 bp and 9 oligos) differs by >0.43 between two conditions indicates a statistically significant change in nucleosome occupancy or position (p<0.05, see Materials and Methods). Accordingly, the decrease in Nuc B occupancy +Dex 1 hr, is highly likely to represent a real change in MMTV chromatin structure. Interestingly, after 4 hrs of Dex treatment the loss of the Nuc B peak is accentuated, accompanied by the appearance of two weaker peaks covering the spacer DNA up and downstream of the normal Nuc B position (green lines, and green Nuc B*1 & B*2 ovals). This novel observation suggests that, rather than being removed, the Nuc B histone octamer is redistributed away from its original location.

Significant, discrete changes in nucleosomes were also evident in the promoter regions of most of the endogenous GR regulated genes on the array. The most common effect was the increase in peak heights for some promoter nucleosome positions after 1 hr dex treatment. This can be seen, for instance, in the region from −400 to +1000 on *MYC* (a dex repressed gene, Fig. 2C). At *HSD11B2* (a gene that requires GR and hSWI/SNF for activation by Dex, [18]), this effect spans 3 kb of the promoter (arrows in Fig. 2D), and was statistically significant for five nucleosome peaks (arrows in Fig. 2E, which shows the traces for repeat –Dex and +Dex 1 hr samples, compare blue and blue-green lines to red and orange lines). Overall, increased nucleosome occupancy at multiple existing peaks was seen on 80% of the GR regulated promoters on the array for which tiling coverage was essentially complete between −2000 and +1000 bp (Fig. 2 & Figs. S4, S5, S6, S7, S8, S9, S10, with the exception of *MMTV*, *SDPR* and *CYP3A4* - Figs. 2A & S5C & S7A). Increases were seen for all peaks within one-to-three kb regions on about half of these promoters (e.g. for *HSD11B2*, *MYC*, *SGK1*, *TSC22D3*, & *PLK2*, Figs. 2C & 2D, and Figs. S4C, S6C & S9C), while the other half showed increases only at a few promoter nucleosomes (e.g. for *PCK1*, *SLC19A2*, & *GEM*, Figs. S4A, S5A & S9A). Note that these increases in measured nucleosome occupancy could potentially result directly from an increased fraction of gene copies covered by histone octamers. Alternatively or in addition, they may reflect some other change in chromatin structure which alters the release of ∼146 bp mononucleosomal fragments by MNase (see Discussion).

Another common effect, seen on 63% of promoters, was the apparent resolution of well-positioned nucleosomes from regions of delocalized nucleosomes. This can be seen, for instance, in the conversion of the broad plateau of nucl/bare ratios of ∼.75 between −1000 and −500 on the *HSD11B2* promoter (without dex) into discrete nucleosome peaks at −900, −800 and −650 after dex addition for 1 hr (compare blue and red lines, Fig. 2D). By contrast, we rarely saw nucleosomes relocalize from discrete positions to new discrete positions at nearby sites (with the formation of flanking peaks associated with the loss of the *MMTV* Nuc B peak, Fig. 2A, being about the clearest example of this rare type of effect).

Intriguingly, most of the changes seen after 1 hour of dex treatment were reversed at 4 hours of dex treatment (green lines in Figs. 2C & 2D, and in Figs. S4, S5, S6, S7 & S9–S10, A & C). Indeed, only a relatively small fraction of the changes seen at 1 hr +Dex were observed to either persist or be accentuated after 4 hrs +Dex (e.g. *MMTV* NucB, Fig. 2A). This indicates that there is a temporal progression of dex-dependent remodeling effects, in which some of the most dramatic early changes revert to normal at later time points.

### Role of hSWI/SNF in GR-driven chromatin changes

The ATP-dependent chromatin remodeling complex, hSWI/SNF, is an essential GR coactivator. Of the two remodeling ATPase subunits present in human cells (BRG1 and the less abundant hBRM), studies have shown that BRG1 is sufficient to coactivate through GR [19], [26]. Thus, to discern the role of hSWI/SNF in GR- and Dex-dependent chromatin changes, we knocked down BRG1 in UL3 cells using siRNA oligos to BRG1. Unlike the control oligos, the specific siRNA reduced BRG1 levels by ∼75% (Fig. 1D), and greatly reduced the response of the *MMTV*-luc construct to Dex (Fig. 1E, dark grey bars). Knock down and control cells were treated with or without dexamethasone for 1 hr., and chromatin harvested. Mononucleosomal fragments were then isolated and used to probe the custom arrays. We found that BRG1 knock down –Dex and +Dex 1 hr curves most frequently fell in between the control –Dex and +Dex 1 hr curves, indicating that hSWI/SNF function might be important for both the low nucleosome occupancy without hormone and the high nucleosome occupancy resulting from 1 hr dex treatment (e.g. in the −900 to −600 region of *HSD11B2*, Fig. 2F). Occasionally, BRG1 knock down caused dramatic increases or decreases in the occupancy of specific nucleosomes, in either –Dex, +Dex 1 hr, or both conditions, that were not seen in the RNAi control cells under either condition (e.g. the strong peak on *HSD11B2* at −100 for BRG1 kd –Dex; also at arrows in Figs. S4, S5, S6, S7, S8, S9, S10, S11, S12, S13, S14). Interestingly, these particularly-strong knock down effects tended to be localized near transcription start sites (of the ten strongest differences observed between −2 and +1 kb on mapped promoters, seven of them were localized between −250 and +250 bp relative to the TSS).

### Use of a PCR-based MNase footprint assay to validate and extend array observations

To confirm and extend results from the microarrays, we used an MNase footprint PCR assay with two sets of primers targeting each of the *MMTV* Nuc B, Nuc D, or Nuc F sequences (arrows in Figure 2A). UL3 cells were untreated or treated with Dex for 20 minutes, 1 hr or 4 hrs, before chromatin isolation, MNase digestion and isolation of ∼146 bp mononucleosomal fragments to serve as templates for PCR. Consistent with the microarray results in Fig. 2A, mononucleosomal products at Nuc B decreased after 20 mins and remained below baseline levels at 1 hr and at 4 hrs (Figure 2B, showing fold change in PCR signal relative to -Dex). We have found that hSWI/SNF can convert two adjacent nucleosomes into altered dinucleosomes (or altosomes), which have an unusual ∼200 bp footprint (intermediate between mono- and dinucleosome footprints [56], [57]). Accordingly, one possible explanation for the decrease, +Dex, of Nuc B signal from isolated ∼146 bp mononucleosomal fragments could be that hSWI/SNF (recruited to MMTV by GR) might convert Nuc B and one of its neighbors into an altosome. However, when we tested this possibility, using the same PCR primers to measure the abundance of *MMTV* Nuc B, D and F regions in isolated ∼200 bp MNase fragments, we found that altosome levels also decrease at Nuc B after 1 or 4 hour dex treatment (Fig. S15). Accordingly, the loss of Nuc B signal does not appear to be due to altosome formation.

### Comparison across genes reveals unexpected general effects of GR & SWI/SNF

**Figure 3:**
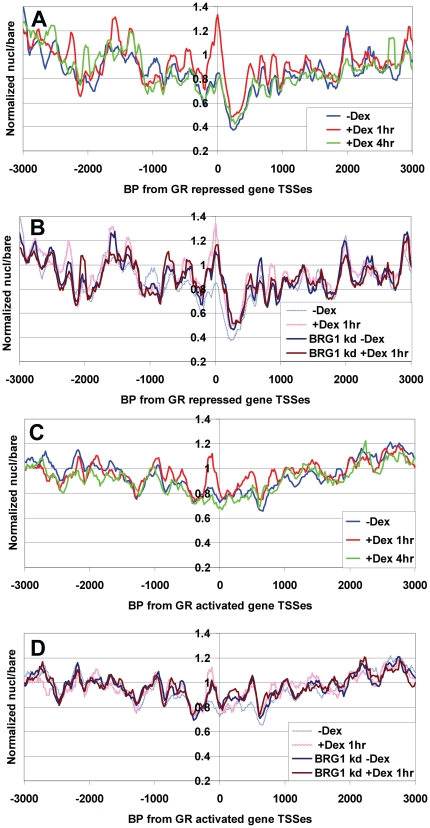
Average nucleosome occupancy rises transiently +Dex surrounding TSSes of GR activated and GR repressed genes. (**A**) & (**B**) Average nucleosome density relative to the transcription start sites of the four Dex repressed genes on the array: *GEM*, *PLK2*, *POMC* & *MYC*. (**C**) & (**D**) Average nucleosome density vs. TSSes for nine of the GR-activated endogenous genes on the array: *HSD11B2*, *TSC22D3*, *PCK1*, *ZBTB16*, *SDPR*, *SGK1*, *SLC19A2*, *CYP3A4* & *SRGN. MMTV* was excluded from this analysis to avoid contributions of non-*MMTV* vector and luciferase sequences upstream of ∼−1200 and downstream of ∼+100). (A) & (C) Comparison of UL3 cells treated with No Dex, Dex 1 hr and Dex 4 hr, as indicated. (B) & (D) Comparison of No Dex and Dex 1 hr conditions in BRG1 knock down and siRNA control cells.

**Figure 4:**
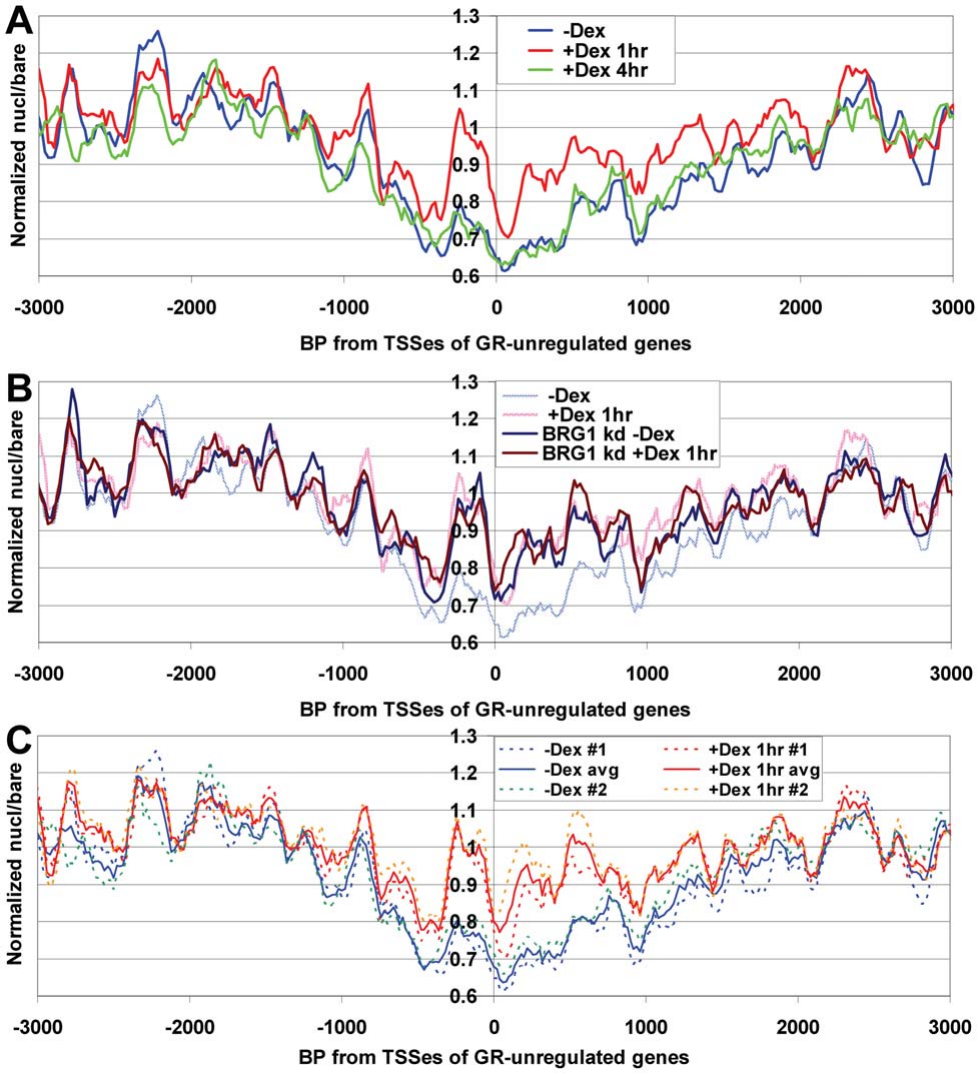
1 hr Dex treatment increases nucleosome occupancy at GR-unregulated promoters. (**A**) & (**B**) As per Fig. 3, but showing average normalized nucl/bare ratios for all GR-independent genes on the array (*CCNA1, CCNB2, CCND1, CCNE1, CD44, CDK1, CDKN1A, CSF1, E2F1, GAPDH, UGT1A6 & UGT1A8*). (**C**) –Dex or +Dex 1 hr results from biological replicate samples (dotted lines) and average from both samples (solid lines).

Because our microarray results provide nucleosome position information across ten GR-activated genes, four GR-repressed genes and twelve GR-independent genes, we were also able to look for systematic changes across all genes in each class. Surprisingly, for all three classes, we found low average nucleosome occupancy around TSSes without Dex that increased significantly after 1 hr Dex treatment (Figs. 3A & 3C and 4A, compare blue and red lines). This effect, measured over the 2 kb region from −1000 to +1000 bp, was greatest for non-regulated genes, intermediate for Dex down-regulated genes and less dramatic, but still significant, for Dex up-regulated genes (p<.0002 in each case). Interestingly, this effect was largely lost after 4 hr Dex treatment, for all three classes of promoters (Figs. 3A, 3C and 4A, green lines), suggesting that Dex-bound GR causes a rapid and transient rise in nucleosome occupancy over promoters that it regulates as well as promoters that it does not.

Because this finding was so unexpected, we performed several tests to rule out possible sources of systematic bias. First, we quantitated our MNase digestion gels to show that digestion levels varied little between conditions and that minor differences in MNase digestion did not correlate with altered promoter nucleosome occupancy (Fig. 1B and Fig. S1, as also described in Additional Methods). Next, we showed that essentially identical results were seen with biological replicate -Dex and +Dex 1 hr samples (Fig. 4C). Finally, we showed that increased occupancy +Dex 1 hr was observed only at promoter regions and not at other upstream or downstream region having any given nucleosome/bare ratio in the no-Dex control (Fig. S3). This rules out possible systematic biases in hybridization signal or data processing which might have caused, for instance, all regions with low nucleosome/bare ratios in the no-Dex sample (a characteristic of promoters -Dex) to show apparently increased nucleosome occupancy +Dex 1 hr. In summary, the observation of increased promoter nucleosome occupancy +Dex 1 hr is reproducible and cannot be linked to sample variability or any systematic bias, indicating that it reflects a real difference in promoter chromatin structure. In contrast to changes observed surrounding TSSes, no significant changes in nucleosome occupancy were observed at transcription termination sites (Fig. S16).

In BRG1 knock down cells, we found that the average nucleosomal occupancy around the TSSes of both GR-activated and GR-repressed genes (both with and without Dex) was usually intermediate between the -Dex and +Dex 1 hr values that were observed for cells transfected with control siRNA oligos (Figures 3B & 3D). This suggests that hSWI/SNF function is required after Dex addition for increased nucleosome occupancy, and, somewhat counter-intuitively, is also required before GR recruitment, for the maintenance of the low occupancy chromatin state. Unlike the GR-regulated promoters, the promoter nucleosome density after BRG1 knock down +/−Dex was very similar to that in +Dex 1 hr control cells, indicating that BRG1 is primarily required to maintain low nucleosome occupancy in the absence of Dex at these promoters (Fig. 4B).

### Nearby GR binding sites may attenuate a Dex-dependent increase in promoter nucleosome occupancy

**Figure 5:**
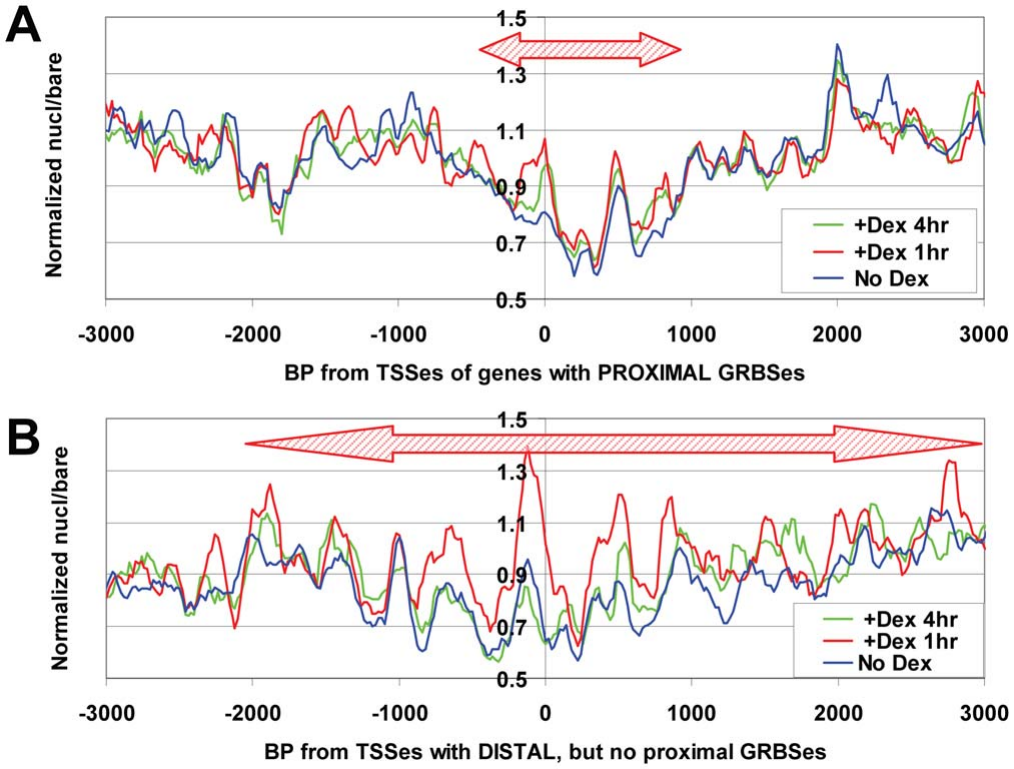
Proximal GRBSes are associated with a lesser increase in nucleosome occupancy after Dex. Nucleosome density relative to TSSes was plotted for either: (**A**) all genes that contain a GR-binding site within 500 bp upstream of the TSS (top panel, *SDPR*, *SLC19A2*, *SRGN*, *GEM* & *POMC* – *MMTV* was excluded because of the presence of luciferase and vector sequences, and lack of knowledge of the insertion site), or (**B**) all genes that lack a promoter proximal GR-binding site, but which have a GR-binding site >1 kb upstream of the TSS (bottom panel, *HSD11B2*, *SGK1*, *TSC22D3* & *PLK2*).

We classified GR-regulated promoters into two groups: five which had well-mapped GR binding sites (GRBSes) in their proximal promoters (the proximal group, with GRBSes from −109 to −400 versus TSSes, with a median value of −304) and four which had no promoter-proximal GRBSes but had well-mapped GRBSes located more than 1 kb upstream of TSSes (the distal group, with GRBSes from −1247 to −19787, with a median value of −1636) [18], [19], [20], [21], [22], [23]. Interestingly, the increase, +Dex 1 hr, in nucleosome occupancy surrounding TSSes was less intense and spanned a 3-fold smaller region for proximal GRBS-group promoters than for distal GRBS-group promoters (compare Fig. 5A and 5B). Moreover, the increase in occupancy over distal GRBS-group promoters was similar in magnitude and breadth to that seen for genes that are not regulated by GR (compare Fig. 5 to Fig. 4A). This suggests the interesting possibility that GR recruitment to promoter proximal sites might regulate transcription by actively suppressing the increase in nucleosome occupancy that occurs at all other Pol II promoters in response to Dex.

To measure of the effect of GR binding on immediately-surrounding chromatin, we plotted nucleosome density relative to all well-mapped GRBSes in two groups: promoter-proximal (within 500 bp of TSSes) and promoter-distal (between −20000 and −500 bp from TSSes), with some genes having GRBSes in more than one group [18], [19], [20], [21], [22], [23]). Nucleosome occupancy was seen to rise +Dex 1 hr in both groups (Fig. S17). Similar results were also seen when we considered the locations of GR binding sites identified in a recent genomewide ChIP-seq study in human lung carcinoma cells [58] (Fig. S18A shows the results for 6 sites within 500 bp of TSSes, and S18B shows results for 5 sites between 1.0 & 2.5 kb from TSSes). Increased occupancy surrounding GRBSes may simply reflect occupancy changes at promoters, since the increases were greatest downstream of GRBSes (towards the promoter) and affected a wider area in the distal group (similar to the results in Fig. 5). Consistent with this, we find that 1 hr Dex treatment had little effect on chromatin surrounding 37 lung carcinoma cell GRBSes that were located >2.5 kb upstream of TSSes [58] (Fig. S18C). We did not see a significant drop in average nucleosome occupancy at or in the vicinity of mapped GR binding sites, arguing against a broad requirement for nucleosome “disruption” to facilitate GR binding. However, the studies which mapped GRBSes by ChIP microarray have an estimated accuracy of > =  +/−200 bp, and this uncertainty regarding specific GR binding positions would make it difficult to detect small localized changes associated with GR binding (e.g. changes in the occupancy of a single nucleosome).

### Genomewide increase in promoter mononucleosome occupancy during T cell activation

**Figure 6:**
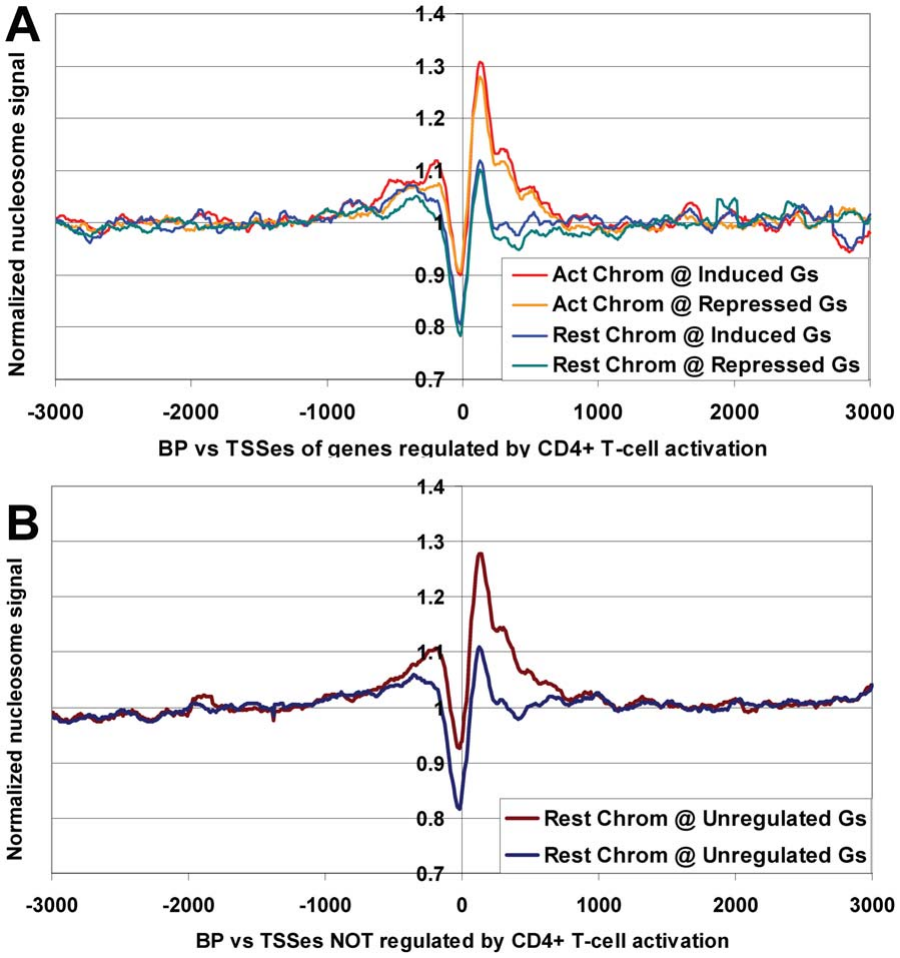
Genomewide increase in promoter nucleosome occupancy upon activation of human CD4+ T cells. Sequence reads from fifty Solexa lanes for resting versus activated human CD4+ T cells [5] were mapped to the human genome, and relative nucleosome frequency versus TSSes determined for genes at least 2-fold induced or at least 2-fold repressed by activation (**A**), or for genes whose expression changed less than 1.5-fold as a result of activation (**B**).

The observation of greatly increased promoter nucleosome occupancy in UL3 cells in response to dex, made us wonder whether similar chromatin changes might occur in response to other stimuli. Furthermore, the fact that similar effects were seen at genes which were regulated or not regulated by dex suggested that these changes in nucleosome occupancy might be occurring on most Pol II genes, genomewide (although this possibility could not be directly confirmed with only the 26 genes on our arrays). To address both of these issues, we examined the data from a recent study which used intensive multiparallel sequencing to compare genomewide nucleosome positions in CD4^+^ T-cells with or without activation by addition of anti-CD3 and anti-CD28 antibodies for 18 hours [5]. Interestingly, Schones et al. noted that the average nucleosome density increased surrounding TSSes of genes that were strongly induced or strongly repressed by T-cell activation. The authors, however, did not examine nucleosome density of genes whose expression was unchanged by T-cell activation. Using the Schones et al. dataset, we compared nucleosome positions on promoters whose expression after T-cell activation was induced by greater than two-fold, repressed by greater than two-fold, or unchanged (altered by less than 1.5-fold). This analysis showed the expected rise in nucleosome density on strongly induced or repressed genes (Fig. 6A), but also revealed an almost identical increase in occupancy on activation-independent genes (Fig. 6B). This same effect was seen for every tested subset of sequencing data from activated versus resting T-cells (over 50 sequencing lanes per condition), and is therefore unlikely to be due to variability in sample preparation, MNase digestion or run-specific biases in sequencing or analysis (Fig. S19B–D). Also, as for our UL3 cell analysis, this effect was only seen at promoters and not at other regions of low nucleosome density (Fig. S19A). Taken together with our observations from UL3 cells, these observations suggest that genomewide changes in nucleosome occupancy near TSSes can occur in response to at least two different stimuli. Furthermore, these changes are unlikely to be artifacts of the nucleosome mapping method, since they can be seen by both microarray and sequencing approaches.

### Increased mononucleosome occupancy during terminal differentiation of human HL60 cells

**Figure 7:**
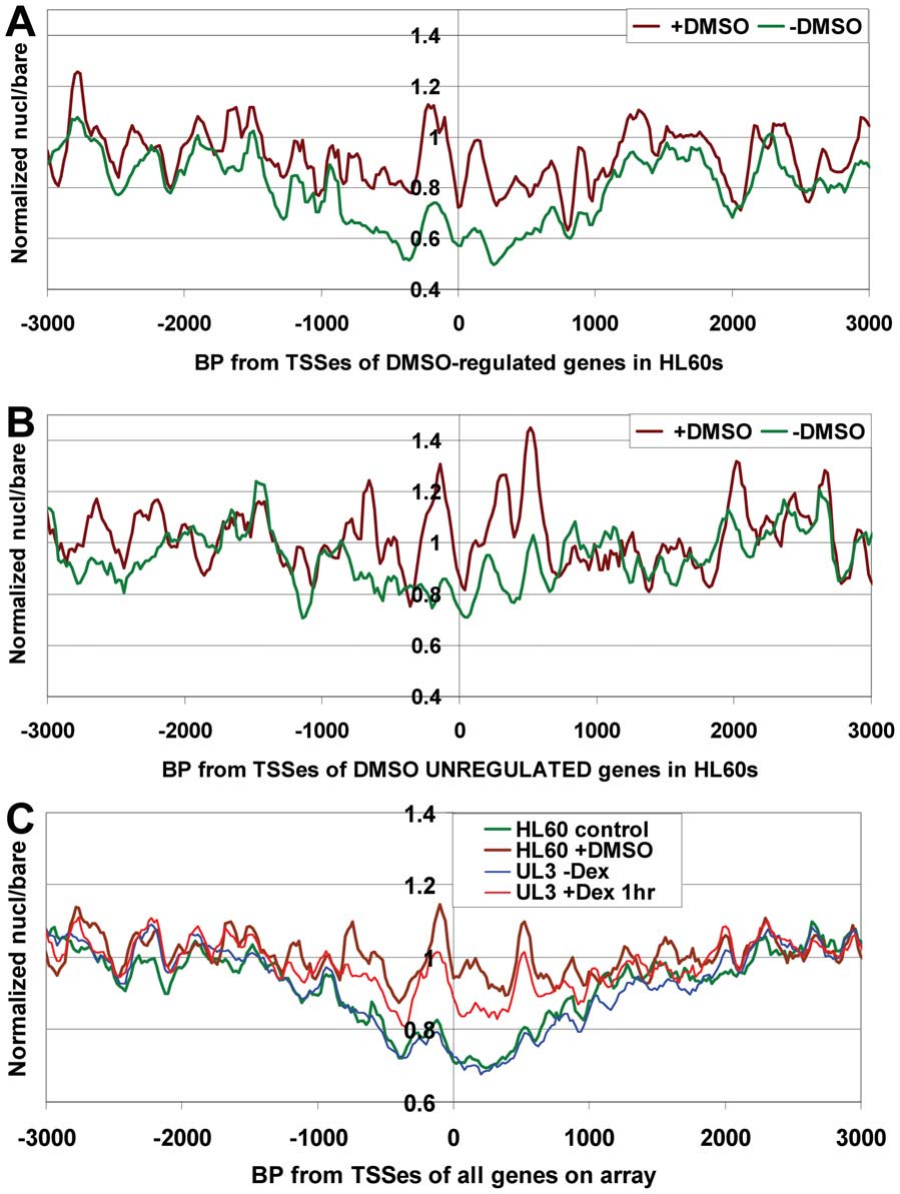
HL60 differentiation to granulocytes is associated with increased promoter nucleosome occupancy. Average quantile-normalized nucleosome occupancy for control HL60 cells versus DMSO-differentiated HL60 cells relative to (**A**) the TSSes of all genes on the array that were significantly up- or down-regulated by 5-day DMSO treatment (up: *MYC*, *CCNE1, CCND1, CCNB2*, *CD44* & *ZBTB16*, down: *GAPDH* & *SGK1*, [59]), or (**B**) all genes on the array that were not regulated by DMSO treatment (showing expression levels well above background and less than a 1.3-fold change with DMSO treatment: *CDK1*, *E2F1*, *CSF1*, *SRGN* & *TSC22D3*, [59]). A similar effect was seen for both up- and downregulated genes, when analyzed separately (data not shown). (**C**) Comparison of average nucleosome densities for control conditions (–Dex for UL3s, –DMSO for HL60s) and after stimulation (+Dex 1 hr for UL3s, +DMSO 5 days for HL60s). Plots showing that increased occupancy +DMSO is only seen for promoter regions (at any given nucl/bare ratio in –DMSO samples) are shown in Fig. S3C & D.

Glucocorticoid response and T-cell activation do not result in a terminally differentiated phenotype or cause cells to exit the cell cycle. To test whether changes in promoter nucleosome occupancy also occur as part of terminal differentiation, we employed our custom tiling microarrays to compare mononucleosome distributions in cycling human HL60 myeloid leukemia cells to those in HL60 cells that had been induced to differentiate into granulocytes by a five-day treatment with DMSO. Granulocytic differentiation of HL60 cells results in downregulation of many genes that promote cell growth and division, including *MYC* (e.g. [52], [59]). As expected, we found that DMSO treatment caused a 3.7-fold decrease in *MYC* mRNA, as measured by RT-PCR. We compared average gene occupancy curves surrounding TSSes of genes shown to be up- or downregulated by 5-day DMSO treatment in HL60 cells to genes that were clearly not regulated by DMSO ([59] GEO accession # GSE14500). Strikingly, we found that treatment of HL60 cells with DMSO caused average mononucleosome levels to rise over a ∼6 kb region surrounding the TSSes of the differentiation-regulated genes on the array, with the greatest effect over the ∼2 kb surrounding the TSSes (Fig. 7A). Furthermore, as was seen in UL3 cells and CD4+ T cells, the same effect was also seen for the genes on the array that were not regulated by DMSO (Fig. 7B). Interestingly, the average nucleosome occupancy patterns relative to TSSes for all genes on the array was essentially identical for both HL60 cells -DMSO and UL3 cells –Dex (Fig. 7C, compare green line to thin blue line). This suggested that the promoter chromatin structures in non-stimulated cycling cell lines of distinct developmental lineages are generally similar, at least as measured by average nucleosome densities near TSSes. Measured nucleosome occupancy was also somewhat similar between HL60+DMSO and UL3 +Dex 1 hr conditions (Fig. 7C, compare brown line to thin red line), suggesting that the chromatin changes resulting from these divergent stimuli are of similar type and magnitude.

## Discussion

At the outset of this study we hypothesized that the recruitment of hSWI/SNF to GR binding sites and GR-regulated promoters might result in discrete changes in nucleosome positions that would activate or repress transcription by allowing or blocking the binding of transcription factors to DNA (as suggested by our recent biochemical studies [60], [61], [62], and for review see [57]). By contrast, in UL3 cells we found almost no clear cases of nucleosomes shifting from one position to another after addition of Dex and/or after knock down of the hSWI/SNF ATPase, BRG1. Instead, the most frequently observed effect, after 1 hr of Dex treatment, was an increase in the apparent occupancy at already existing nucleosome peaks within ∼2 kb of transcription start sites. This effect could be seen for specific nucleosomes on individual promoters, as well as on average across all Dex repressed or Dex activated promoters. Surprisingly, a strong increase in average nucleosome occupancy was also seen over the promoters of genes that were not regulated by Dex, an effect that was reproducible in independent samples and could not be explained by differences in MNase digestion or any systematic bias in the microarray analysis. Accordingly, these results suggest that the most prominent effect of GR and Dex on chromatin is to rapidly increase measured nucleosome occupancy on a large fraction of Pol II promoters, apparently genomewide.

Given that this effect is seen after only 1 hour of dex treatment, it seems unlikely that it would be due to GR-directed transcriptional activation or repression of a second wave of transcription factors. This is also consistent with studies showing that inhibition of translation via cyclohexamide does not alter the distribution of genes that are upregulated, unregulated and downregulated by a two or four hour incubation with Dex [66]. For Dex-unregulated genes, while there are no known or expected GR binding sites near their promoters, the increased nucleosome occupancy that is observed could potentially be mediated by very long range influences, *in cis* or even *in trans*, of GR bound to chromatin. Indeed, recent studies have indicated that more than half of all functional GRBSes are located over 10 kb away from the start site of genes they regulate [21], and GR and Dex can mediate dramatic unfolding of large chromatin domains in fluorescence microscopy studies [69]. Furthermore, all but one of the genes on our array had a start site within 100 kb of one or more of the over 15 thousand GR binding sites recently identified in A549 cells (an average of one per 180 kb [58]). On the other hand, there is growing evidence that hormone bound GR can regulate a variety of cellular kinases and other signaling molecules, independent of its direct transcriptional regulatory functions (e.g. [67]). These altered signal transduction cascades might then regulate common transcription factors, co-activators, basal factors or chromatin modifying complexes that might have a broad, GRBS-site-independent effect on promoter chromatin structure in response to Dex. Interestingly, global as well as gene-specific dephosphorylation of the linker histone H1 has been observed after prolonged Dex exposure [68]. While the slower timing of this effect appears to rule out its direct involvement in the chromatin changes we observe, it does establish a precedent for Dex and GR-dependent changes in fundamental chromatin structure cell-wide.

Interestingly, the increase in apparent promoter nucleosome occupancy that we see at 1 hr of Dex treatment is largely lost after 4 hrs of Dex treatment. This may be most consistent with the possible mechanism described above, in which increased promoter nucleosome occupancy results from activation of cellular kinases by Dex-bound GR, an effect that could potentially be transient, and attenuated by long-duration Dex exposures. This observation is also broadly consistent with other studies indicating that chromatin remodeling effects associated with the activation of specific genes by GR and other nuclear hormone receptors can change over time. For instance, a greater than 24 hr exposure to Dex has been shown to silence *MMTV* transcription and eliminate restriction enzyme accessibility at Nuc B [63]. In addition, studies examining the effect of estradiol-bound estrogen receptor (ER) on the human *PS2* gene showed that, under some circumstances, ER activation could lead to ∼2 hr long periodic cycles of transcription factor binding and release together with promoter nucleosome alterations at the promoter [64]. The possibility of this type of cycling effect was also suggested for GR by a set of *in vitro* transcription studies on chromatin [65].

Using RNAi, we showed that BRG1-containing hSWI/SNF was important for the high nucleosome occupancy after 1 hr dex treatment at GR-repressed and GR-activated promoters, suggesting that part of the increase in nucleosome occupancy at these promoters may be due to GR-dependent recruitment of hSWI/SNF. We also found that hSWI/SNF was essential for the low nucleosome occupancy in the absence of Dex at both GR-regulated and GR-independent genes. This surprising effect might potentially mean that hSWI/SNF is continuously present, and remodeling chromatin, near the TSSes of most genes on the array. Basal levels of hSWI/SNF recruitment might be possible through the dozens of activators, repressors and basal factors that it has been shown to bind to (e.g. [70], [71]). Alternatively, early biochemical characterization of hSWI/SNF indicated that the complex is present at ∼10,000 copies per cell [72], raising the possibility that it might be sufficiently abundant to have significant non-targeted effects on genomic chromatin. The seemingly more likely possibility, however, is that the BRG1 hSWI/SNF complex may be essential for the transcriptional activation or repression of some other factor (be it a histone modifying enzyme, histone chaperone or other remodeling complex) which is required to promote low occupancy over TSSes in cycling cells.

In addition to the effects of Dex in U2OS cells, we also found striking increases in measured nucleosome occupancy near TSSes of both regulated and non-regulated genes in human HL60 cells induced to differentiate to granulocytes (as assayed by tiling microarray), and CD4+ T-cells activated by addition of anti-CD3 and anti-CD28 antibodies (as assayed by Solexa/Illumina multiparallel sequencing, [5]). These results indicate that genomewide alterations in promoter nucleosome occupancy may be a common cellular response to a variety of stimuli.

The simplest interpretation of this effect is that that the fractional occupancy of promoter DNA by histone octamers (e.g. the fraction of gene copies with a nucleosome covering each position on DNA) increases in response to these stimuli, perhaps as the result of new deposition of nucleosomes using S-phase independent chaperones. Intriguingly, one recent study revealed that, unlike the case for yeast promoters, human Pol II promoters have sequence characteristics which are expected to promote higher than average nucleosome occupancy [73]. This suggests the interesting possibility that high promoter nucleosome occupancy is the default state, and that low occupancy must be actively maintained. If so, the stimuli we have examined here might inhibit these active processes, causing promoters to revert to an intrinsic sequence-encoded high occupancy state.

It must be emphasized, however, that apparently increased occupancy could also be caused by other effects that might alter the abundance of nucleosomal MNase fragments from promoter regions in our samples. For instance, apparently low occupancy could result if association with nuclear matrix proteins prevented the release of mononucleosomes after MNase digestion, or if association with heterochromatin proteins, HMGs or variant linker histones blocked digestion between adjacent promoter nucleosomes (resulting in dinucleosome-sized fragments that would be lost when ∼146 bp products mononucleosomal MNase products were isolated (Fig. S1 & [5]). It is also possible that differences in histone tail modifications, linker histones or core histone variants might change the MNase sensitivity of promoter mononucleosomes. In the most extreme case this might result in the complete hydrolysis of octamer-covered DNA by MNase (reducing signal in both assays). Alternatively, if these effects changed the MNase protected footprint size to more than ∼155 bp or less than ∼135 bp, the range we isolated by PAGE (or to a size greatly different from the ∼150 bp band isolated in [5]), these larger or smaller fragments would not be detected. Nevertheless, whether the observed occupancy increase is due to increased histone octamer abundance or to one of these other effects, the observations described here provide evidence for a striking, unanticipated change in chromatin structure associated with Pol II promoter DNA, apparently genomewide, which can be caused by at least three different inducing conditions.

What possible function might be ascribed to genomewide increases in promoter nucleosome occupancy in response to environmental stimuli? HL60 cell differentiation and T-cell activation are long-term processes that result in dramatic changes in cellular functioning. In cases like these, the system-wide reduction in promoter accessibility by increased promoter nucleosome occupancy might function to globally slow the production of proteins that promote and regulate default cellular processes involved in undifferentiated growth. At the same time, condition-specific transcription factors and signaling cascades would be expected to be able to contend with this general effect at promoters, allowing the production of new proteins specific for the differentiated or activated cell's functions.

For GR-regulated genes, a global increase in promoter nucleosome density could potentially act to suppress plieotropic responses that might result from GR and Dex activation of non-genomic signal transduction cascades (such as phosphorylation of transcription factors by src kinase, as reviewed in [67]). Even though this effect is transient, and lost after 4 hours of dex treatment, it might be sufficient to limit transcriptional responses to glucocorticoid hormone more specifically to genes containing GRBSes or containing DNA binding sites for second-wave transcription factors regulated by GR. In this regard, it was quite interesting to note that the rise in nucleosome occupancy was of lower intensity and lesser range around TSSes within 500 bp of a GRBS. This suggests that nearby GR binding may be able to suppress the increase in promoter nucleosome occupancy that is otherwise stimulated, genomewide, by Dex. If so, the maintenance of these promoters in a state of low nucleosome occupancy during this initial period after Dex addition may be an essential aspect of their regulation by Dex. It is unclear, as yet, whether the transient increase in promoter chromatin density after Dex addition quantitatively alters transcription rate, and it will be interesting, in future studies, to see whether this is the case, using techniques capable of directly measuring transcription rate such as nuclear run on, GRO-seq or ChIP microarray analysis of Pol II occupancy [5], [74], [75].

## Materials and Methods

### Cell culture and RNAi

UL3 cells (generated as described in [46]) were grown essentially as per [28], [46], using media containing 10% charcoal/dextran treated FBS. In order to maintain the stable expression of GR and *MMTV*-luc, the media also contained 500 µg/mL G418, and 1 µg/mL puromycin. For dexamethasone (dex) treatment, cells were split into phenol red free media 48 hours before harvest, dexamethasone (1000x stock in ethanol) was added to a final concentration of 10 nM. Control cells received ethanol alone. For BRG1 knock down experiments, cells were plated at a density of 2.95×10^4^/cm^2^ and transfected 24 hrs later with 1.95 pmol/cm^2^ of BRG1 dsRNA (Santa Cruz) or control dsRNA(Santa Cruz) using oligofectamine (Invitrogen), essentially as per [76]. Cells were treated with dexamethasone for the indicated times, and harvested 72 hours after transfection. Human HL-60 cells (ATCC #CCL-240) were grown essentially as per [52], and induced to differentiate into granulocytes by the addition of 1.5% DMSO to the media for 5 days.

### Luciferase Assays, mRNA measurement & Western Blotting

Cells were harvested using trypsin, and washed in ice cold PBS. Cell lysates were prepared for luciferase measurement according to manufacturer instructions (Promega), with the inclusion of 1X Protease Inhibitor Cocktail (Pierce) in the lysis buffer. Luminescence units were normalized to protein concentration as measured by BCA protein assay (Bio-Rad). For Western blotting, PBS-washed cells were resuspended in RIPA lysis buffer with 1X Protease Inhibitor Cocktail (Pierce), lysed by freeze/thawing, and 10 µg of each sample resolved by 8% SDS-PAGE before transfer to PVDF membrane. Anti BRG1 rabbit polyclonal [72] or anti α-actin rabbit polyclonal (Cell Signaling) primary antibodies were used at 1∶2000 dilution, followed by goat anti-rabbit HRP secondary antibody (Jackson Laboratories) at 1∶15,000 dilution, and incubation with ECL reagent (Promega). mRNA from cells treated with Dex for various times were reverse transcribed, the cDNA subjected to RT-PCR (see Additional Methods, Text S1, for primers), products separated by electrophoresis and quantitated using Molecular Dynamics IQ tools. Values were normalized to the signal for *GAPDH*.

### Chromatin Isolation and MNase digestion

PBS-washed cells were resuspended in 11.3 µL per cm^2^ plate surface area in ice cold lysis buffer (10 mM Tris pH 7.4, 10 mM NaCl, 3 mM MgCl_2_, 0.5% NP40), and chromatin collected by microcentrifugation at 1600 RPM for 15 min. at 4°C. Chromatin pellets were resuspended in 200 µL per 10^7^ cells in storage buffer (50 mM Tris pH 7.4, 40% glycerol, 5 mM MgCl_2_, 0.1 mM EDTA), flash frozen in liquid N_2_, and stored at −80°C until use. Concentration of DNA in chromatin samples was measured by dilution into 2 M NaCl, and spectrophotometry at 260 nm. 150 µg of DNA was incubated at 30°C with 212 U MNase in a volume of 100 µL reaction buffer (50 mM Tris pH 7.4, 25 mM KCl, 2.5 mM CaCl_2_, 5 mM MgCl_2_, 12.5% glycerol) for 4 min. The reactions were stopped by adding 100 µL stopping buffer (50 mM Tris pH 7.4, 2% SDS, 100 mM EDTA, 200 mM NaCl). 15 µL of proteinase K (Promega, 20 mg/mL) was added and samples were incubated at 60°C for 2 hrs. Samples were extracted with an equal volume of phenol, treated for 1.5 hrs at 37°C with 18 µg RNAse (Qiagen), phenol extracted again, ethanol precipitated in the presence of 40 µg glycogen carrier, and separated by 4% PAGE. Bands corresponding with mononucleosome sized-fragments (∼146 bp) and altosome-sized fragments (∼200 bp) were excised from the gel and eluted in TE at 4°C with continuous shaking overnight. There were no evident differences in MNase digestion level or isolated band sizes for any sample or condition (Fig. 1B and Fig. S1).

### MNase Footprint PCR Assay

The concentration of each MNase footprint fragment was measured by quantitation of ethidium bromide signal in PAGE gels relative to standard DNAs, using Molecular Dynamics IQ tools, and 5 ng of each template was used in 25 µL PCR reactions, products were separated by 5% PAGE, and the ethidium bromide signal quantitated. See Additional Methods, in Text S1, for primer sequences.

### Tiling Microarray Analysis

Mononucleosomal MNase footprint fragments from UL3 or HL60 cells (a total of 1 to 4 µg) were isolated as described above and used to probe custom Nimblegen tiling arrays covering 25 gene loci, with a resolution of ∼10 bp and bearing two forward and two reverse oligos for each sequence (see tables S1 and S2; human genomic sequences based on hg18 build). Overall, the array contains about 320,000 oligos spanning 800,000 bp. MNase digested bare genomic DNA fragments of average ∼500 bp length served as controls for variability in hybridization efficiency. Data from of the four sets of oligos on the array (forward 1/F1, forward 2/F2, reverse 1/R1 and reverse 2/R2) were separately normalized to a median value of 1.0. For each oligo set (e.g. all F1s), we took the ratio of normalized nucleosomal signal over the average normalized signal for two separate control bare DNA runs. By separately analyzing each primer set, we were able to remove one class of common hybridization artifact, in which both forward oligos gave very different signal strengths from both reverse oligos (Fig. S2A). The median nucl/bare ratio for all four oligo sets (F1, F2, R1, R2) was then calculated. Next, to allow the comparison between the results from different microarrays, we used a quantile normalization method (frequently used to in comparisons of microarray datasets [6], [77] (see Additional Methods, in Text S1, for details). This improved the fit between biological repeat datasets (e.g. Fig. S2C). Lastly, to reduce high frequency noise (aberrant peaks of signal much smaller than an ∼146 bp nucleosome), we used a deviation-weighted exponential smoothing function that de-emphasizes positions where the four oligos for that position give very divergent results (see Additional Methods, in Text S1, for details & Fig. S2D). To assess the significance of observed changes, we used the average absolute difference between repeat –Dex and +Dex 1 hr samples to establish an error estimate for each position. This estimate was used to structure t-Tests which took into account the number of oligos in an assayed region (9 oligos for a nucleosome position) and the number of tests performed (see Additional Methods, in Text S1). The raw and processed data from this study are MIAME compliant, and available on the NCBI Gene Expression Omnibus (http://ncbi/nlm.nih.gov/geo), with the accession number GSE25281.

### Determination of average nucleosomal signal relative to TSSes or GRBSes

Quantile normalized nucleosome/bare ratios for all oligonucleotides whose centers fell within 20 kb of a list of referenced genomic positions (TSSes or GRBSes) were placed into 20 bp bins, based on their position and orientation relative to the reference position, and averaged. For ease of representation, the data was smoothed using a 5-bin sliding window. To determine whether the difference between +dex 1 hr and no dex values for the 2 kb surrounding TSSes in binned data was significantly greater than for other 2 kb regions, average standard deviation for all 2 kb regions between −19 and +19 kb was estimated using the repeat dex 1 hr and no dex samples, and this was used in t-Tests comparing average (dex1 hr – nodex) values for the −1 to +1 kb region to all 18 other regions. Nucleosome end reads for activated and control CD4+ T-cells (.fastq format, [5], SRA000234) were assigned to human genomic locations (hg18) using Bowtie [78]. Only reads that mapped to a single, unique genomic position with less than two mismatches were used. Frequency maps for nucleosome positions were then generated from each aggregate dataset, counting 146 bp from the start of each sequence read to the end of the nucleosome, with a 10 bp bin size. Data was then normalized to an average of 1. Gene expression microarray data (an average of two runs for activated or non-activated CD4+ T cells, [5], GEO GSE10437) was used to compute activated/non-activated expression ratios. All genes with a ratio of > = 2 were considered to be induced by activation (6073 total), those with a ratio of < = 0.5 were considered to be repressed by activation (8030 total), and those with ratios between 1.5 and 0.67 were considered to be unchanged by T-cell activation (12531 total). Genomic locations and orientations for the start sites of each gene set (from the UCSC genome browser) were then used to determine nucleosome frequency vs. TSSes, essentially as for the microarray mapping data.

## Supporting Information

Text S1 **Additional methods.** This file includes: 1) all primer sequences used for MNase-footprint PCR, 2) additional details on methods used for normalization and smoothing of microarray data, estimation of nucleosome occupancy, and statistical analyses, 3) additional details on tests which argue against possible systematic biases in the data, and 4) references for papers cited in this file and/or in Table S1.
(DOC)

Figure S1 **MNase digestion differences cannot account for dex effects on chromatin.** (**A** & **B**) Ethidium bromide stain of gels used to isolate mononucleosomal & altosomal MNase fragments. BP positions (based on NEB 50 bp and PhiX174/HaeIII ladders) are indicated on the left. (**C**) Gel A after isolation of mono, inter and dinucleosomal bands. (**D**) Quantitation of gel lanes with ImageQuant shows very little variability in MNase digestion. Note that there was little variability in MNase digestion between samples, which were all digested to >60% mononucleosome level. Furthermore, the modest differences in MNase digestion that do exist do not correlate with increased/decreased nucleosome occupancy (e.g. the BRG1_KD samples, digested to ∼64% mononucleosomes, showed promoter nucleosome occupancy intermediate between the low and high extremes seen for control –Dex and +Dex 1hr samples, which were both digested to ∼73% mononucleosomes). See also Additional Method in Text S1.
(TIF)

Figure S2 **Analytical methods for nucleosome mapping by microarray.** (**A**) Example of F1, F2, R1 and R2 raw data, showing raw cy3 signal from MNase digested bare DNA at *HSD11B2*. Note how F1 and F2 track together, but often differ from R1 and R2. (**B**) Curves showing median-normalized nucleosome/bare signal versus percentile in dataset for each UL3 cell experiment. The average curve (black line) was used as the standard for quantile normalization. (**C**) Comparison of simple median normalization (top) versus quantile normalization (bottom) for the two independent –Dex samples at the GAPDH promoter. In both cases the data was smoothed as described in (D). (**D**) Example of deviation weighted exponential smoothing. Note how high peaks in the unsmoothed quantile-normalized data (brown line) correspond to regions where the standard error of the median between F1, F2, R1 and R2 for that position are high. Making use of this information in the smoothing function (red line) removes noise peaks that are not effectively removed using normal exponential smoothing (dotted orange line).
(TIF)

Figure S3 **Increased nucleosome occupancy only occurs near TSSes and is not due to hybridization bias.** We wished to know whether the increase of nucleosome occupancy surrounding transcription start sites (TSSes) with 1hr Dex treatment of UL3s and with DMSO treatment of HL60s was specific for promoter regions or was also true for other regions of low nucleosome density. In **A** & **C**, we tested for this type of systematic bias by calculating the “Dex effect” for each oligo on the array, where Dex effect =  (normalized nucl/bare Dex1hr) -(normalized nucl/bare Nodex). Similarly, for HL60 cells, we calculated the DMSO effect: (nucl/bare +DMSO) – (nucl/bare control). We then split the data into two groups, “proximal” for all oligos w/in 2kb of a TSS and “distal” for all oligos more than 2kb away from a TSS. For each nucl/bare ratio under the control condition (-Dex or –DMSO) on the x axis, we then plotted the average Dex or DMSO effect on the y axis. If, for example, there was a hybridization artifact on our +DMSO array that gave an aberrantly high signal at all low-occupancy regions, this would result in a high DMSO effect at nucl/bare ratios less than 1 for both proximal and distal oligos. Instead, increased nucleosome occupancy +Dex1hr or +DMSO was only seen for proximal/promoter regions (blue lines). Furthermore, this was true for oligos showing both low and high occupancy under control conditions (indicating increased occupancy both in troughs as well as at nucleosomal peaks). In **B** & **D**, we plotted the fraction of all oligos showing any given nucl/bare ratio (y) versus nucl/bare ratio (x), comparing proximal and distal groups for each treatment condition. Note how the treatment (+DMSO or +Dex 1hr) and control (-DMSO or –Dex) curves for the distal oligos are precisely superimposable (compare red and green curves), indicating no systematic effect of treatment on the distribution of nucleosome occupancies in these regions. By contrast, for proximal oligos, the no treatment curves (blue) are shifted left relative to the treatment curves (yellow), reflecting increased nucleosome occupancy after treatment. Note, A & B show the average curves resulting from the all pairwise combinations of the repeated Nodex and Dex1hr samples. Similar results were also seen for each individual pair of Nodex and Dex1hr arrays.
(TIF)

Figure S4 **GR activated genes:** ***PCK1*** **&** ***SGK1*** **.** Promoter nucleosome density of the GR activated genes *PCK1/PEPCK* (A) & (B), and *SGK1* (C) & (D). Figs. S4, S5, S6, S7, S8, S9, S10, S11, S12, S13, S14 show plots of quantile normalized nucleosome/bare ratios for all GR-regulated genes for which densely tiled oligos covered more than 60% of the -2000 to +1000 promoter region (all but SRGN) and most other genes on the array (all but CCNB2, UGT1A6 & UGT1A8). A ratio of 1.0 corresponds to ∼50% nucleosome occupancy and a ratio of 2.1 corresponds to ∼100% nucleosome occupancy (see Fig. 2 legend, or Additional Methods for details). (A) & (C) show -Dex, +Dex 1hr and +Dex 4hr. (B) & (D) show -Dex & +Dex 1hr from control cells (dotted lines) or BRG1 knock down cells (solid lines). The arrow in (B) highlights a BRG1 dependent effect that differs from both + and - Dex control cells.
(TIF)

Figure S5 **GR activated genes:** ***SLC19A2*** **&** ***SDPR*** **.** Promoter nucleosome density of the GR activated genes *SLC19A2* (A) & (B), and *SDPR* (C) & (D), as described in Figure S4. (A) & (C) show -Dex, +Dex 1hr and +Dex 4hr. (B) & (D) show -Dex & +Dex 1hr from control cells (dotted lines) or BRG1 knock down cells (solid lines).
(TIF)

Figure S6 **GR activated genes:** ***ZBTB16*** **&** ***TSC22D3*** **.** Promoter nucleosome density of the GR activated genes *ZBTB16/PLZF* (A) & (B), and *GILZ/TSC22D3* (C) & (D), as described in Figure S4. (A) & (C) show -Dex, +Dex 1hr and +Dex 4hr. (B) & (D) show -Dex & +Dex 1hr from control cells (dotted lines) or BRG1 knock down cells (solid lines). The arrows in (B) & (D) highlight BRG1 dependent effects that differ from both + and - Dex control cells.
(TIF)

Figure S7 **GR activated gene** ***CYP3A4*** **and control gene** ***GAPDH*** **.** Promoter nucleosome density for the GR activated gene *CYP3A4* (A) & (B) and the GR- and hSWI/SNF-unregulated gene *GAPDH* (C) & (D), as described in Figure S4. (A) & (C) show -Dex, +Dex 1hr and +Dex 4hr. (B) & (D) show -Dex & +Dex 1hr from control cells (dotted lines) or BRG1 knock down cells (solid lines).
(TIF)

Figure S8 **GR-activated** ***MMTV*** **promoter.** (**A**) Same as Fig. 2A (reproduced here for comparison). Note that the relative weakness of Nuc C, and the split nature of Nucs D through F, is consistent with microarray mapping results for an MMTV construct integrated into another cell line (MDA-kb2 cells, Dennis et al. (2007) *Genome Res* 17:928-39), and may also be consistent with indirect end-labeling results which sometimes showed multiple local peaks of nuclease sensitivity in the gaps between Nucs C through Nucs E (e.g. Trotter & Archer (2004) *Mol Cell Biol* 24:3347-58, Truss et al. (1995) *EMBO J* 14:1737-51, and Richard-Foy & Hager (1987) *EMBO J* 6:2321-28). Importantly, indirect end-labeling identifies positioned nucleosomes by the appearance of relatively strong MNase digestion sites flanking ∼146 bp regions of relative protection. These conditions can occur regardless of the fraction of gene copies that bear a positioned nucleosome. Thus, indirect end labeling is expected to provide information about nucleosome edges but will not provide accurate information about relative nucleosomal occupancy. For instance, while the *c-myc* promoter Nucleosomes 7 and 8 (identified by indirect end labeling) did appear as discrete nucleosome position peaks in our analysis, the relative weakness of these peaks suggests that these positioned nucleosomes are present on only a subset of gene copies (Fig. 2C). (**B**) Effects of BRG1 knock down on MMTV promoter chromatin. Dotted lines show nucleosome density maps -Dex & +Dex 1hr from control cells (from A) and solid lines show –Dex and +Dex 1hr results from BRG1 knock down cells. Note that NucB decreases +Dex 1hr in the BRG1 knock down as well as control cells (an effect that was confirmed by MNase footprint PCR on mononucleosomal fragments, which showed that 1hr dex treatment of BRG1 knock down cells caused Nuc B occupancy to decrease to 57 +/- 16% of –Dex levels, similar to the results from control cells in Fig. 2B). This was somewhat unexpected, given that prior studies showed that introduction of BRG1 into cells that lack it allows Dex-induced restriction enzyme accessibility at NucB (Trotter KW & Archer TK (2004) *Mol Cell Biol* 24:3347-58). However, the ability of hSWI/SNF to activate *MMTV* transcription does not perfectly correlate with its ability to increase NucB accessibility. For instance, mutations in the BAF60a hSWI/SNF subunit which reduce GR-hSWI/SNF interactions decreased *MMTV* activation by Dex by 10-fold, but only decreased NucB accessibility by two-fold (Hsiao PW et al. (2003) *Mol Cell Biol* 23:6210-20). This suggests that the low levels of BRG1 that remain after siRNA transfection in our studies, or the generally minor alternative ATPase hBRM, may be sufficient to disrupt NucB but not sufficient to make other chromatin changes at *MMTV* which are required for maximal transactivation.
(TIF)

Figure S9 **GR repressed genes:** ***GEM*** **&** ***PLK2*** **.** Promoter nucleosome density of the GR repressed genes *GEM* (A) & (B), and *PLK2/SNK* (C) & (D), as described in Figure S4. (A) & (C) show -Dex, +Dex 1hr and +Dex 4hr. (B) & (D) show -Dex & +Dex 1hr from control cells (dotted lines) or BRG1 knock down cells (solid lines). The arrows in (B) & (D) highlight BRG1 dependent effects that differ from both + and - Dex control cells. In (A) and (C), previously-mapped GR binding sites are indicated by squares.
(TIF)

Figure S10 **GR repressed genes:** ***POMC*** **&** ***MYC*** **.** Promoter nucleosome density of the GR repressed genes *POMC* (A) & (B), and *MYC* (C), as described in Figure S4. (A) shows -Dex, +Dex 1hr and +Dex 4hr for POMC. For +Dex 4hr results on myc, see Fig. 2C. (B) & (C) show -Dex & +Dex 1hr from control cells (dotted lines) or BRG1 knock down cells (solid lines).
(TIF)

Figure S11 **Non-GR regulated cell cycle control genes:** ***CCNA1*** **&** ***CCND1*** **.** Promoter nucleosome density of the GR-unregulated genes *CCNA1/cyclin A1* (A) & (B), and *CCND1/cyclin D1* (C) & (D), as described in Figure S4. (A) & (C) show -Dex, +Dex 1hr and +Dex 4hr. (B) & (D) show -Dex & +Dex 1hr from control cells (dotted lines) or BRG1 knock down cells (solid lines). The arrow in (B) highlights a BRG1 dependent effect that differs from both + and - Dex control cells.
(TIF)

Figure S12 **Non-GR regulated cell cycle control genes:** ***CCNE1*** **&** ***CDK1*** **.** Promoter nucleosome density of the GR unregulated genes *CCNE1/cyclin E1* (A) & (B), and *CDK1/CDC2* (C) & (D), as described in Figure S4. (A) & (C) show -Dex, +Dex 1hr and +Dex 4hr. (B) & (D) show -Dex & +Dex 1hr from control cells (dotted lines) or BRG1 knock down cells (solid lines).
(TIF)

Figure S13 **Non-GR regulated cell cycle control genes:** ***CDKN1A*** **&** ***E2F1*** **.** Promoter nucleosome density of the GR unregulated genes *CDKN1A/p21* (A) & (B), and *E2F1* (C) & (D), as described in Figure S4. (A) & (C) show -Dex, +Dex 1hr and +Dex 4hr. (B) & (D) show -Dex & +Dex 1hr from control cells (dotted lines) or BRG1 knock down cells (solid lines). The arrow in (B) highlights a BRG1 dependent effect that differs from both + and - Dex control cells.
(TIF)

Figure S14 **Non-GR regulated, hSWI/SNF-regulated genes:** ***CSF1*** **&** ***CD44*** **.** Promoter nucleosome density of the GR-independent, hSWI/SNF activated genes *CSF1* (A) & (B), and *CD44* (C) & (D), as described in Figure S4. (A) & (C) show -Dex, +Dex 1hr and +Dex 4hr. (B) & (D) show -Dex & +Dex 1hr from control cells (dotted lines) or BRG1 knock down cells (solid lines). The arrow in (B) highlights a BRG1 dependent effect that differs from both + and - Dex control cells. The green line in (B) shows data from Ozsolak et al. 2007, Nat Biotechnol 25:244-8 (GEO accession # GSE6385).
(TIF)

Figure S15 **Loss of Nuc B mononucleosomes is not due to altered dinucleosome formation by hSWI/SNF.** (**A**) Mononucleosome levels at *MMTV* NucF, NucD and NucB at the indicated times, as measured by MNase footprint PCR (repeated from Fig. 2B, to facilitate comparison). (**B**) Altered dinucleosomes formed by hSWI/SNF (altosomes) have an unusual ∼200 bp MNase footprint size, which allowed us to assay altosome levels on *MMTV* by subjecting ∼200 bp MNase-resistant chromatin fragments to MNase footprint PCR. The results showed that altosome levels at Nuc B did not rise significantly after 20 mins or 1 hour of dex treatment, and actually decreased at 4 hrs, arguing against the hypothesis that NucB is converted into altosomes. Interestingly, an increase in altosome formation was observed at Nuc F and D at 1 hr., which moved towards baseline levels after 4 hr. Because the footprint size of altosomes cannot arise from any arrangement of normal nucleosomes, and because no other remodeling complex is known to form altosomes, this observation suggests that hSWI/SNF affects nucleosomes up to ∼1 kb beyond its expected sites of recruitment (the GRE elements located within Nuc B). A return of altosomes to baseline levels at 4 hr is consistent with altosomes' innate tendency to revert to normal nucleosomes. (**C**) Prior studies indicate that, the partial agonist RU486 induces GR binding to *MMTV* and recruitment of hSWI/SNF, but does not support the recruitment of other coactivators, including pCIP, SRC1, and p300 (Fryer CJ et al. (2000) *J Biol Chem*, 275:17771-7), allowing the examination of hSWI/SNF effects independent of other GR coactivators. Consistent with these studies, we find that RU486 decreases the amount of *MMTV* reporter induction compared to dexamethasone (to 76 +/- 6 fold after 24hrs, as compared to 513 +/- 27 fold with Dex). When mononucleosome positions were analyzed by MNase footprint PCR for UL3 cells treated with RU486, we found that reduction of Nuc B occupancy was apparent after 20 min. and was increased at 1 and at 4 hrs, similar to dex treatment. (**D**) Altosome levels at upstream nucleosomes D and F also peaked at 1 hr. and declined by 4 hr. Intriguingly, altosomes at Nuc B were elevated throughout the RU486 time course, unlike dexamethasone treatment. The data may suggest that altosomes are formed at Nuc B by hSWI/SNF, but that the other coactivators recruited by Dex-bound GR, or the higher resulting transcription rate, cause them to be removed. In addition, the observation that altosome levels remain high after 4hrs of RU486 treatment only at NucB implies either continued localized hSWI/SNF action or localized stabilization of this product. Accumulation of altosomes at NucB may help to explain why a RU486 treatment was less effective than Dex at inducing restriction enzyme accessibility at NucB after 1 hr (Mymryk JS & Archer TK (1995) *Mol Endocrinol* 9:1825-1834).
(TIF)

Figure S16 **Dex treatment does not significantly alter nucleosome density at transcription termination sites.** As for Fig. 5A, but looking at average nucleosome density relative to transcription termination sites (ends of transcribed regions), for all endogenous genes for which nucleosome positions were mapped at least 5 kb downstream of termination sites.
(TIF)

Figure S17 **Remodeled chromatin extends further downstream of distal GR binding sites.** (**A**) Average nucleosome occupancy mapped relative to GR binding sites that map to within 500 bp upstream of the TSS of their regulated genes (locations relative to TSSes: SDPR ∼-50, SRGN ∼-300, SLC19A2 ∼-150, GEM ∼-320, and POMC ∼-400). (**B**) Average nucleosome occupancy relative to GR binding sites greater than 1kb upstream of regulated TSSes (locations relative to TSSes: HSD11B2 ∼1500, SDPR ∼19800, SGK1 ∼-1290, TSC22D3∼1700, and GEM ∼1770, PLK2 ∼1250 & ∼ -2700). See Table S1 for details.
(TIF)

Figure S18 **Changes in nucleosome occupancy relative to GR binding sites in human A549 lung cells.** As for Fig. S17, but looking at average nucleosome density relative to subsets of GR binding sites identified in human A549 lung carcinoma cells (Reddy et al. 2009, Genome Res 19:2163-71). (**A**) all six GR binding sites within 500 bp of TSSes. (**B**) all five GR binding sites between 1 and 2.5 kb of TSSes. (**C**) all 37 GR binding sites covered by our array and at least 6kb from TSSes. Dotted lines show the results from repeat samples. Solid lines show the average result.
(TIF)

Figure S19 **Increased nucleosome occupancy with CD4+ T-cell activation is not due to general bias.** **A**) We examined the nucleosome count data from Schones et al. using a similar metric to that used for our microarray data in Fig. S3. Briefly, we mapped the treatment effect (acttivated_nucl_counts/avg_activated_nucl_counts) – (resting_nucl_counts/avg_resting_nucl_counts) versus normalized nucleosome density from the resting data (resting_nucl_counts/avg_resting_nucl_counts) for all nucleosome count data within 500 bp of TSSes (proximal) or all data more than 500 bp from any known TSS (distal). We found that a strong increase in nucleosome density was only seen at promoters (blue line) and not in distal regions (red line). In contrast to our microarray results, there was a distinct tendency towards a weak positive effect at nucleosome count values less than 1.0 (below average) and an increasing negative effect at values greater than 1.0 (the negative slope of red line). This would be expected based on the discrete count nature of the sequencing data. For example, when inherent sampling variability in sequencing gives a reads/avg_reads value that is higher than the actual value for one sample, a second sample is likely to yield a reads/avg_reads value that is closer to the actual value. This would result in some degree of apparent negative treatment effect at high reads/avg values, and positive effect at low values. Importantly, even though the strong treatment effect at promoters goes away at nucl_count/avg_nucl_count ratios greater than ∼1.5, positions with these read values make up 75-80% of all reads within the proximal (yellow curve) and distal (green curve) regions. Hence increased nucleosome occupancy is specifically seen around TSSes at the ∼75% of locations where nucl/avg_nucl values are below ∼1.5x genomic average. **B-D**) The Schones et al data consists of more than 50 Illumina sequencing lanes per condition that appear to have been derived from several distinct samples. To determine whether variability in sample preparation could have caused the observed increased occupancy at TSSes with T-cell activation, we compared average nucleosome density around TSSes for the whole data set, resting (dotted lines) or activated (solid lines) to that for the first three and last three lanes for each dataset (SRR#s 711-713 and 840-842 for resting, and 749-751 and 803-805 for activated). The results for TSSes of genes upregulated (**B**), downregulated (**C**), or unaffected (**D**) by T-cell activation showed that, while some variability is evident between samples, this cannot account for the great increase in nucleosome density around all three classes of TSSes with T-cell activation.
(TIF)

Table S1 **Genes covered by the nucleosome mapping Nimblegen arrays.** Blue: genes activated by GR and Dex. Pink: genes repressed by GR and Dex. Grey: cell cycle control genes that are not regulated by GR and Dex in U20S cells. For citations given in the table, see Additional Methods, in Text S1.
(DOC)

Table S2 **Genomic locations and accession numbers for genes on the array.** All coordinates are relative to the HG18 human genome build. TSS: transcription start site.
(DOC)
