# Supplementary material for: Multiple Distinct Stimuli Increase Measured Nucleosome Occupancy around Human Promoters
Source: PLoS One. 2011 Aug 11;6(8):e23490. doi: 10.1371/journal.pone.0023490 (PMC3154950; doi:10.1371/journal.pone.0023490)
Supplement: Text S1 — Additional methods. This file includes: 1) all primer sequences used for MNase-footprint PCR, 2) additional details on methods used for normalization and smoothing of microarray data, estimation of nucleosome occupancy, and statistical analyses, 3) additional details on tests which argue against possible systematic biases in the data, and 4) references for papers cited in this file and/or in Table S1. (DOC) [file pone.0023490.s001.doc]

**ADDITIONAL METHODS**

**Primers used for RT-PCR**

*SGK1*, forward 5’-atctcgcacctgaggtgctt-3’

*SGK1*, reverse 5’-gcaggccatacagcatctcat-3’

*GEM*, forward 5’-agtacagccaccgcaaccgc-3’

*GEM*, reverse 5’-ggccagccgccgttcattct-3’

*CD44*, forward 5’-agaaggtgtgggcagaagaaaa-3’

*CD44*, reverse 5’-cattctgcaggttccttgtct-3’

*MYC*, forward 5’-gggtttatctaactcgctgtag-3’

*MYC*, reverse 5’-gagtcgtagtcgaggtcatagt-3’

*GAPDH*, forward 5’-gtcagtggtggacctgacct-3’

*GAPDH*, reverse 5’-tgagcttgacaaagtggtcg-3’

**Primers used for MNase Footprint PCR at *MMTV***

NucF, forward 1(f1) 5’-TCTGCTGCAAACTTGGCATA-3’

NucF, reverse 1(r1) 5’-AGAGTCAAGGGTGAGAGCCC-3’

NucF, forward 2 (f2) 5’-AGGGCTCTCACCCTTGACTC-3’

NucF, reverse 2 (r2) 5’-AGGTCGAGTTCTCCGAATTG-3’

NucD, f1 5’-ATAGGAGACAGGTGGTGGCA-3’

NucD, r1 5’-ATGGTAAGGGGGCATCTGTT-3’

NucD, f2 5’-TACAGACCAACAGATGCCCC-3’

NucD, r2 5’-GCCATTGACTGTAACCCACC-3’

NucB, f1 5’-GAACATTATTCTGCAAAAACTTATGG-3’

NucB, r1 5’-TACTTAAGCCTTGGGAACCG-3’

NucB, f2 5’-CGGTTCCCAAGGCTTAAGTA-3’

NucB, r2 5’-CCAAACCAAGTCAGGAAACC-3’.

**Normalization of microarray data**

Examining the raw microarray data, we found that the intensity profile for MNase digested and sonicated bare DNA probes (each with an average length of ~500 bp) were very similar, indicating that neither control DNA introduces significant error due to preferential cleavage of some positions by either MNase or sonication. However, when we used 146 bp size-selected bare DNA fragments, this resulted in serious disparities between sonicated and MNase digested bare DNA signals (probably because fragments this small after sonication or MNase digestion represent only the subset of DNAs that are most susceptible to fragmentation by each method).

We noted that the curves of normalized nucleosome/bare ratios versus percentile rankings (where the median equals 50%) could differ significantly between data sets (Fig. S2B). This indicated that differences in background signal as well as hybridization response could distort nucleosome occupancy curves and prevent direct comparison across samples. To address this issue we used a quantile normalization procedure that is commonly used for gene expression microarray data [1], and that was effective in allowing comparison between nucleosome position datasets from tiling microarrays [2]. Briefly, normalized nucleosome/bare values were ranked from lowest to highest, and tables of values at each percentile across seven array experiments were used to generate a single table of average values. Each dataset was then quantile normalized such that the value at a given percentile in the ranked data was set to equal the value for the same percentile in the average curve. This normalization method was effective at improving the fit of the nucleosomal datasets derived from the same condition (nodex 1 & 2 and dex1hr 1 & 2), reducing average absolute difference for values at each position from 0.31 to 0.28. It also clearly improved the fit between nucleosomal traces (for example, see Fig. S2C).

**Deviation-weighted smoothing of nucleosome position data**

Previous studies using tiling microarrays to map human nucleosome positions have shown a moderately high level of high frequency noise (sharp signal differences between adjacent oligos separated by only ~10 bp) [2]. This noise can be reduced, but not eliminated by using multiple forward and reverse oligos for each sequence [3]. Because nucleosomes have 146 bp footprints, it is reasonable to apply a smoothing function to the data, so long as the smoothed interval is small relative to the size of a nucleosome. We chose to use an exponential-decay smoothing function that weighted all oligos within 40 bp of a central reference oligo by 0.5^((bp to reference oligo)/10)^. In addition, given the nucleosome/bare ratios for each of the four oligo spots on the array (F1, F2, R1 & R2) and the median value, we could calculate a deviation from the median value for each position. This value divided by the average deviation from the median for all positions on the array provides a measure of reliability for each median value (higher when this value is low). Incorporating this reliability score resulted in the following smoothing function:

where σ_y_ is the deviation from the median value for F1, F2, R1 & R2 oligos for position y, and σ_avg_ is the average deviation from the F1, F2, R1 & R2 median for all positions on the array. We found that this smoothing function performed much better than linear smoothing, and was also better than standard exponential smoothing at removing anomalous single-oligo peaks (Fig. S2D).

**Estimates of absolute nucleosome occupancy**

Since each point on the nucleosome occupancy curve corresponds to the center of a 50 bp oligo, a peak of data points corresponding to a well-positioned nucleosome will not cover the full 146 bp footprint of the mononucleosome. This is because all 50 bp of each oligo must overlap the mononucleosome fragment to give a signal. Accordingly, a perfectly positioned nucleosome will hybridize with only ~9 oligos spaced by 10 bp ( ^(146bp-50bp)^/_10bp_ = 9.6). This same effect makes low value linker regions between well-positioned nucleosomes seem longer (covering a number of oligos equal to ^(linker length + oligo length^)/_10bp_). To estimate the nucleosome/bare value that corresponds to 100% nucleosome occupancy, we considered an idealized case where well positioned 146 bp nucleosomes are all separated by 60 bp of linker DNA (thought to be typical of mammalian interphase cells). In this case, for each 206 bp, 96 BP would give max signal and 110 bp would give zero. With a dataset normalized to a median value of 1.0, the signal for an oligo covered by a well-positioned nucleosome would be 1.0*^206^/_96_, or ~2.1. Satisfyingly, we find that the highest nucleosomal peaks tend to be very close to this value. Accordingly, we estimate that locations with nucleosome/bare values of ~2.1 indicate 100% nucleosome occupancy, while values of ~1 indicate 50% occupancy, etc.

We noted that the shapes of the nucleosome peaks were almost always rounded. This is in contrast to yeast nucleosome positioning studies [4,5,6,7,8], where nucleosome peaks mapped by tiling microarray or next generation sequencing frequently appeared as plateaus separated by sharp canyons, indicating the presence of precisely positioned nucleosomes. The exponential smoothing function we used would be expected to smooth sharp edges locally (~+/10 or 20 bp), but does not fundamentally alter peak shape (as can be seen, e.g., in Supplemental Fig. S2D). It is also possible that nucleosomal fragments which only overlap a tiled oligo by ~40 bp could give partial signal, resulting in a blurred nucleosomal edge. Neither of these effects, however, could convert the ~146-bp signal plateau that would arise from a perfectly and invariantly positioned nucleosome into a gradually rounded peak. Instead, since similarly rounded peaks predominate in other studies of human nucleosome positions (using both microarrays [2] and direct sequencing [9]), we are inclined to think that the rounded peaks we observe indicate a certain looseness in nucleosome positioning in human cells – such that regions of strong nucleosome occupancy are characterized by several overlapping positions clustered around a central most-favored position. Some possibilities for why human chromatin displays this apparent sloppiness while yeast chromatin does not might include longer average linker regions or the presence, on most human nucleosomes, of linker histone H1.

**Statistical Analyses**

Error in quantile normalized nucleosome/bare ratios was estimated by measuring the absolute difference between values for repeat samples under identical conditions (no dex #1 & #2 and dex 1hr #1 & #2), averaged across all oligos on the arrays. For the non-smoothed data, this value was 0.281, which corresponds to a standard deviation of 0.198. Removal of high frequency noise using deviation weighted exponential smoothing reduced the standard deviation to 0.140. On the array, a perfectly positioned nucleosome will give full signal over ~9 oligos (nine 50mer oligos, spaced by 10 bp, spanning 146 bp). Accordingly, for a change in a single nucleosome’s position or occupancy to be significant, the difference in average nucleosome/bare signal for all 9 oligos should give a t statistic sufficient to reach p<.05, where t = (mean_1_-mean_2_)/sqrt(2*0.140^2^/9). In cases where multiple tests were performed, the required t-statistic was adjusted using the conservative Bonferroni method (look-up_p_value = target_p_value/#_of_tests). Considering all nucleosome positions across all promoters from -2kb to +1kb (~7200 total tests), a difference in means of 0.43 was estimated to be significant at p<.05. This was consistent with manual analysis of a subset of the data (testing whether nucleosome peaks showing mean differences between dex 1hr and nodex of >= 0.43 from experiment#1 displayed the same direction of effect in experiment#2, e.g. Fig. 2E). This same general approach was used to determine the significance of changes in larger areas (adjusting n, or number of oligos, and number of tests accordingly). This showed, for instance, that treatment with dex for 1 hr results in a significant rise in nucleosome occupancy from +1kb to -1kb surrounding the *HSD11B2* TSS (p < .0001, considering all 400 2kb regions tiled on the array as repeated assays in the t-Test).

**Increased nucleosome occupancy over promoters does not result from any systematic bias in the datasets.**

We performed several tests to ensure that the increased nucleosome occupancy seen surrounding transcription start sties in UL3 cells +Dex 1hr, HL60 cells +DMSO and T-cells upon activation was not due to artifacts of sample preparation or data analysis. First, one recent study [8] showed that direct sequence mapping data for yeast nucleosome positions could be distorted if MNase digestion yielded less than 40% mononucleosomes (as compared to MNase digestion yielding mostly or completely mononucleosomes). To confirm that this was not an issue in our study, we quantitated lanes from our preparative gels used to isolate MNase products (Fig. S1). These results showed that effects of differential MNase digestion were unlikely to alter mapping results, since our mononucleosome percentages were high and relatively constant (between 63 and 73%).

Next, we reasoned that it could be possible for an artifact of sample preparation or array hybridization to give, for instance, a systematic, artificially high signal at regions of low nucleosome occupancy in one sample but not another. Since it is well established that Pol II TSSes, on average, have low nucleosome occupancy [2,4,5,6,8,9,10], this would result in an apparent increase in promoter nucleosome occupancy in samples suffering from this artifact. While it was unlikely that this type of effect would occur in repeated Dex 1hr samples and not in repeated Nodex samples, we nonetheless, wanted to further rule this out. To do so, we plotted the “treatment response” (measured occupancy with treatment minus occupancy without) versus no-treatment occupancy values, for sites near TSSes versus sites far from TSSes. The results, described in detail in Fig. S3 A & C, indicate that increased occupancy with treatment (+Dex 1hr for UL3 cells, and +DMSO for HL60 cells) occurs only near TSSes, and at all values of no-treatment occupancy. Furthermore, while TSS-proximal regions show a distinct shift in the frequency of nucleosome/bare values with treatment (from a low average curve –Dex or –DMSO (blue lines in Fig. S3 B & D) to high values +Dex 1hr or +DMSO (yellow lines), there was no change in the frequency curves with treatment at TSS-distal regions (green & red lines). For both of these measures, similar results were seen for quantile normalized data (Fig. S3) and median normalized data (not shown).

Schones et al. stated that they used MNase-digested nucleosomal fragments that were ~80% mononucleosomal for their direct sequencing studies, but did not provide further information about individual samples [9]. To test whether variation in MNase digestion or other aspects of their sample preparation might have contributed to the observed increase in TSS-proximal nucleosome occupancy with T-cell activation, we tested whether subsets of their data (the first three and last three sample lanes, out of ~50 for each condition) produced the same effect. As shown in Fig. S19 B-D, while there was some variability between sample subsets, both early and late subsets showed low TSS-proximal nucleosome occupancy in resting cells and high occupancy in activated cells, at promoters upregulated (B), downregulated (C) or non-regulated (D) by activation, indicating that this effect is not caused by sample preparation artifacts. Next, similar to our microarray analysis, we mapped “treatment effect” versus nucleosome occupancy at TSS-proximal versus TSS-distal locations. We found that strongly-increased nucleosome occupancy with activation was only seen at TSS-proximal locations (Fig. S19 A). Note that the reduction of this effect at nucleosome occupancy values greater than 1.5-fold average, representing less than 25% of all values, is likely to be due to the stochastic nature of direct sequencing (see legend for further discussion). In summary, for both our microarray analysis and the Schones et al. sequencing data, we find no evidence to suggest that systematic biases in the data from different samples can explain the observed increase of nucleosome density around TSSes with treatment.

**References for Additional Methods**

1. Pradervand S, Weber J, Thomas J, Bueno M, Wirapati P, et al. (2009) Impact of normalization on miRNA microarray expression profiling. Rna 15: 493-501.

2. Ozsolak F, Song JS, Liu XS, Fisher DE (2007) High-throughput mapping of the chromatin structure of human promoters. Nat Biotechnol 25: 244-248.

3. Dennis JH, Fan HY, Reynolds SM, Yuan G, Meldrim JC, et al. (2007) Independent and complementary methods for large-scale structural analysis of mammalian chromatin. Genome Res 17: 928-939.

4. Yuan GC, Liu YJ, Dion MF, Slack MD, Wu LF, et al. (2005) Genome-scale identification of nucleosome positions in S. cerevisiae. Science 309: 626-630.

5. Segal E, Fondufe-Mittendorf Y, Chen L, Thastrom A, Field Y, et al. (2006) A genomic code for nucleosome positioning. Nature 442: 772-778.

6. Albert I, Mavrich TN, Tomsho LP, Qi J, Zanton SJ, et al. (2007) Translational and rotational settings of H2A.Z nucleosomes across the Saccharomyces cerevisiae genome. Nature 446: 572-576.

7. Narlikar L, Gordan R, Hartemink AJ (2007) A nucleosome-guided map of transcription factor binding sites in yeast. PLoS Comput Biol 3: e215.

8. Weiner A, Hughes A, Yassour M, Rando OJ, Friedman N (2010) High-resolution nucleosome mapping reveals transcription-dependent promoter packaging. Genome Res 20: 90-100.

9. Schones DE, Cui K, Cuddapah S, Roh TY, Barski A, et al. (2008) Dynamic regulation of nucleosome positioning in the human genome. Cell 132: 887-898.

10. Ioshikhes IP, Albert I, Zanton SJ, Pugh BF (2006) Nucleosome positions predicted through comparative genomics. Nat Genet 38: 1210-1215.

11. Trotter KW, Archer TK (2004) Reconstitution of glucocorticoid receptor-dependent transcription in vivo. Mol Cell Biol 24: 3347-3358.

12. Hebbar PB, Archer TK (2007) Chromatin-dependent cooperativity between site-specific transcription factors in vivo. J Biol Chem 282: 8284-8291.

13. Wang JC, Derynck MK, Nonaka DF, Khodabakhsh DB, Haqq C, et al. (2004) Chromatin immunoprecipitation (ChIP) scanning identifies primary glucocorticoid receptor target genes. Proc Natl Acad Sci U S A 101: 15603-15608.

14. So AY, Chaivorapol C, Bolton EC, Li H, Yamamoto KR (2007) Determinants of cell- and gene-specific transcriptional regulation by the glucocorticoid receptor. PLoS Genet 3: e94.

15. Sugatani J, Nishitani S, Yamakawa K, Yoshinari K, Sueyoshi T, et al. (2005) Transcriptional regulation of human UGT1A1 gene expression: activated glucocorticoid receptor enhances constitutive androstane receptor/pregnane X receptor-mediated UDP-glucuronosyltransferase 1A1 regulation with glucocorticoid receptor-interacting protein 1. Mol Pharmacol 67: 845-855. Epub 2004 Nov 2022.

16. Stafford JM, Wilkinson JC, Beechem JM, Granner DK (2001) Accessory factors facilitate the binding of glucocorticoid receptor to the phosphoenolpyruvate carboxykinase gene promoter. J Biol Chem 276: 39885-39891.

17. Trotter KW, Fan HY, Ivey ML, Kingston RE, Archer TK (2008) The HSA domain of BRG1 mediates critical interactions required for glucocorticoid receptor-dependent transcriptional activation in vivo. Mol Cell Biol 28: 1413-1426.

18. Galliher-Beckley AJ, Williams JG, Collins JB, Cidlowski JA (2008) Glycogen synthase kinase 3beta-mediated serine phosphorylation of the human glucocorticoid receptor redirects gene expression profiles. Mol Cell Biol 28: 7309-7322.

19. Rogatsky I, Wang JC, Derynck MK, Nonaka DF, Khodabakhsh DB, et al. (2003) Target-specific utilization of transcriptional regulatory surfaces by the glucocorticoid receptor. Proc Natl Acad Sci U S A 100: 13845-13850.

20. Bilodeau S, Vallette-Kasic S, Gauthier Y, Figarella-Branger D, Brue T, et al. (2006) Role of Brg1 and HDAC2 in GR trans-repression of the pituitary POMC gene and misexpression in Cushing disease. Genes Dev 20: 2871-2886.

21. Chi TH, Wan M, Lee PP, Akashi K, Metzger D, et al. (2003) Sequential roles of Brg, the ATPase subunit of BAF chromatin remodeling complexes, in thymocyte development. Immunity 19: 169-182.

22. Wang X, Nagl NG, Wilsker D, Van Scoy M, Pacchione S, et al. (2004) Two related ARID family proteins are alternative subunits of human SWI/SNF complexes. Biochem J 383: 319-325.

23. Nagl NG, Jr., Patsialou A, Haines DS, Dallas PB, Beck GR, Jr., et al. (2005) The p270 (ARID1A/SMARCF1) subunit of mammalian SWI/SNF-related complexes is essential for normal cell cycle arrest. Cancer Res 65: 9236-9244.

24. Nagl NG, Jr., Zweitzig DR, Thimmapaya B, Beck GR, Jr., Moran E (2006) The c-myc gene is a direct target of mammalian SWI/SNF-related complexes during differentiation-associated cell cycle arrest. Cancer Res 66: 1289-1293.

25. Nagl NG, Jr., Wang X, Patsialou A, Van Scoy M, Moran E (2007) Distinct mammalian SWI/SNF chromatin remodeling complexes with opposing roles in cell-cycle control. Embo J 26: 752-763.

26. Zhang ZK, Davies KP, Allen J, Zhu L, Pestell RG, et al. (2002) Cell cycle arrest and repression of cyclin D1 transcription by INI1/hSNF5. Mol Cell Biol 22: 5975-5988.

27. Dunaief JL, Strober BE, Guha S, Khavari PA, Ålin K, et al. (1994) The retinoblastoma protein and BRG1 form a complex and cooperate to induce cell cycle arrest. Cell 79: 119-130.

28. Kang H, Cui K, Zhao K (2004) BRG1 controls the activity of the retinoblastoma protein via regulation of p21CIP1/WAF1/SDI. Mol Cell Biol 24: 1188-1199.

29. Lee D, Kim JW, Seo T, Hwang SG, Choi EJ, et al. (2002) SWI/SNF complex interacts with tumor suppressor p53 and is necessary for the activation of p53-mediated transcription'. J Biol Chem 277: 22330-22337.

30. Oruetxebarria I, Venturini F, Kekarainen T, Houweling A, Zuijderduijn LM, et al. (2004) P16INK4a is required for hSNF5 chromatin remodeler-induced cellular senescence in malignant rhabdoid tumor cells. J Biol Chem 279: 3807-3816. Epub 2003 Nov 3806.

31. Roberts CW, Orkin SH (2004) The SWI/SNF complex--chromatin and cancer. Nat Rev Cancer 4: 133-142.

32. Zhang HS, Gavin M, Dahiya A, Postigo AA, Ma D, et al. (2000) Exit from G1 and S phase of the cell cycle is regulated by repressor complexes containing HDAC-Rb-hSWI/SNF and Rb-hSWI/SNF. Cell 101: 79-89.

33. Liu R, Liu H, Chen X, Kirby M, Brown PO, et al. (2001) Regulation of csf1 promoter by the swi/snf-like baf complex. Cell 106: 309-318.

34. Hendricks KB, Shanahan F, Lees E (2004) Role for BRG1 in cell cycle control and tumor suppression. Mol Cell Biol 24: 362-376.

35. Liu H, Mulholland N, Fu H, Zhao K (2006) Cooperative activity of BRG1 and Z-DNA formation in chromatin remodeling. Mol Cell Biol 26: 2550-2559.

36. Vicent GP, Nacht AS, Smith CL, Peterson CL, Dimitrov S, et al. (2004) DNA Instructed Displacement of Histones H2A and H2B at an Inducible Promoter. Mol Cell 16: 439-452.

37. Fan HYT, K. W.Archer, T. K.Kingston, R. E. (2005) Swapping function of two chromatin remodeling complexes. Mol Cell 17: 805-815.
