# Supplementary figures and images for: Multiple Distinct Stimuli Increase Measured Nucleosome Occupancy around Human Promoters
Source: PLoS One. 2011 Aug 11;6(8):e23490. doi: 10.1371/journal.pone.0023490 (PMC3154950; doi:10.1371/journal.pone.0023490)

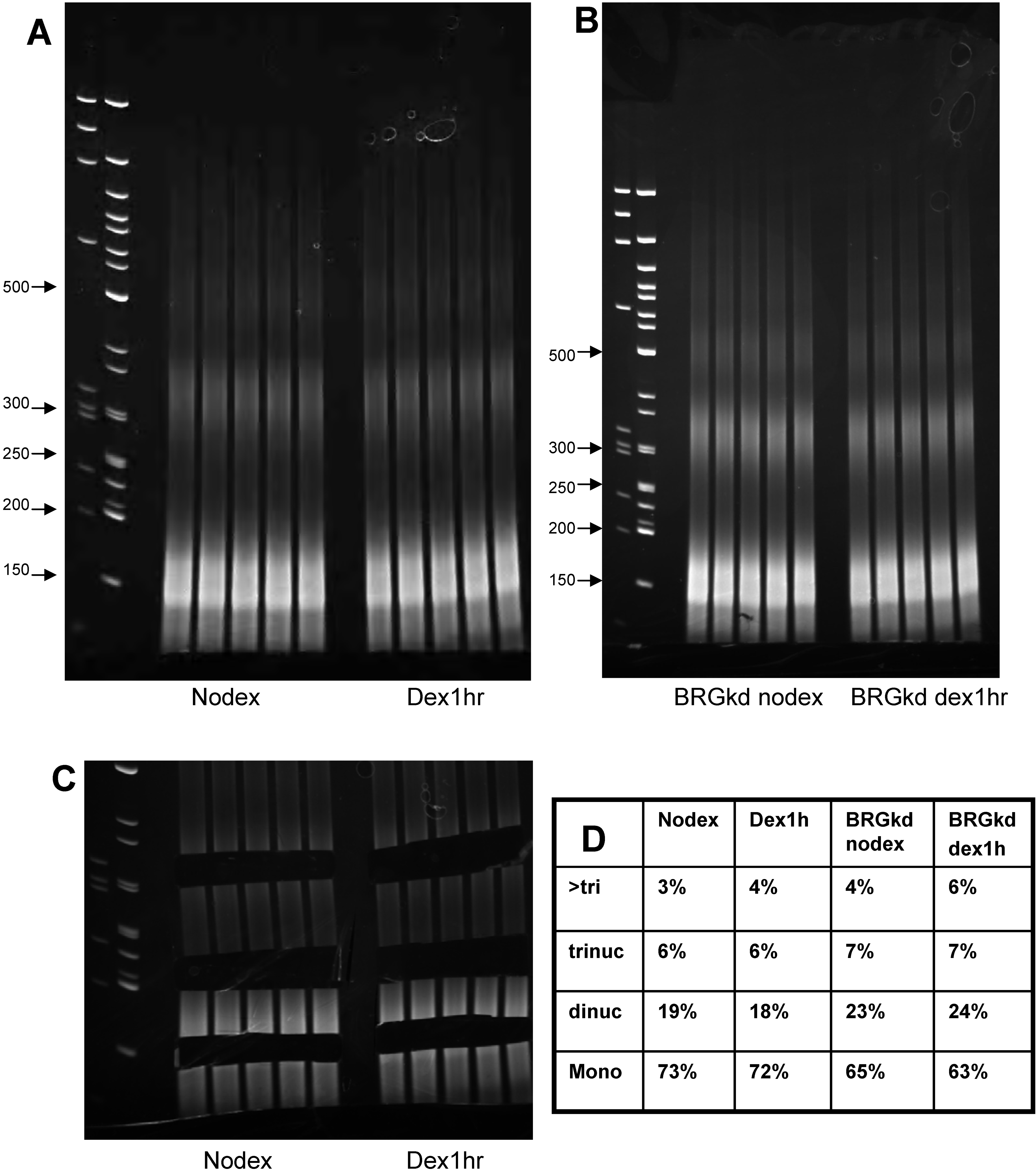

Supplement: Figure S1 — MNase digestion differences cannot account for dex effects on chromatin. (A & B) Ethidium bromide stain of gels used to isolate mononucleosomal & altosomal MNase fragments. BP positions (based on NEB 50 bp and PhiX174/HaeIII ladders) are indicated on the left. (C) Gel A after isolation of mono, inter and dinucleosomal bands. (D) Quantitation of gel lanes with ImageQuant shows very little variability in MNase digestion. Note that there was little variability in MNase digestion between samples, which were all digested to >60% mononucleosome level. Furthermore, the modest differences in MNase digestion that do exist do not correlate with increased/decreased nucleosome occupancy (e.g. the BRG1_KD samples, digested to ∼64% mononucleosomes, showed promoter nucleosome occupancy intermediate between the low and high extremes seen for control –Dex and +Dex 1hr samples, which were both digested to ∼73% mononucleosomes). See also Additional Method in Text S1. (TIF) [file pone.0023490.s002.tif]

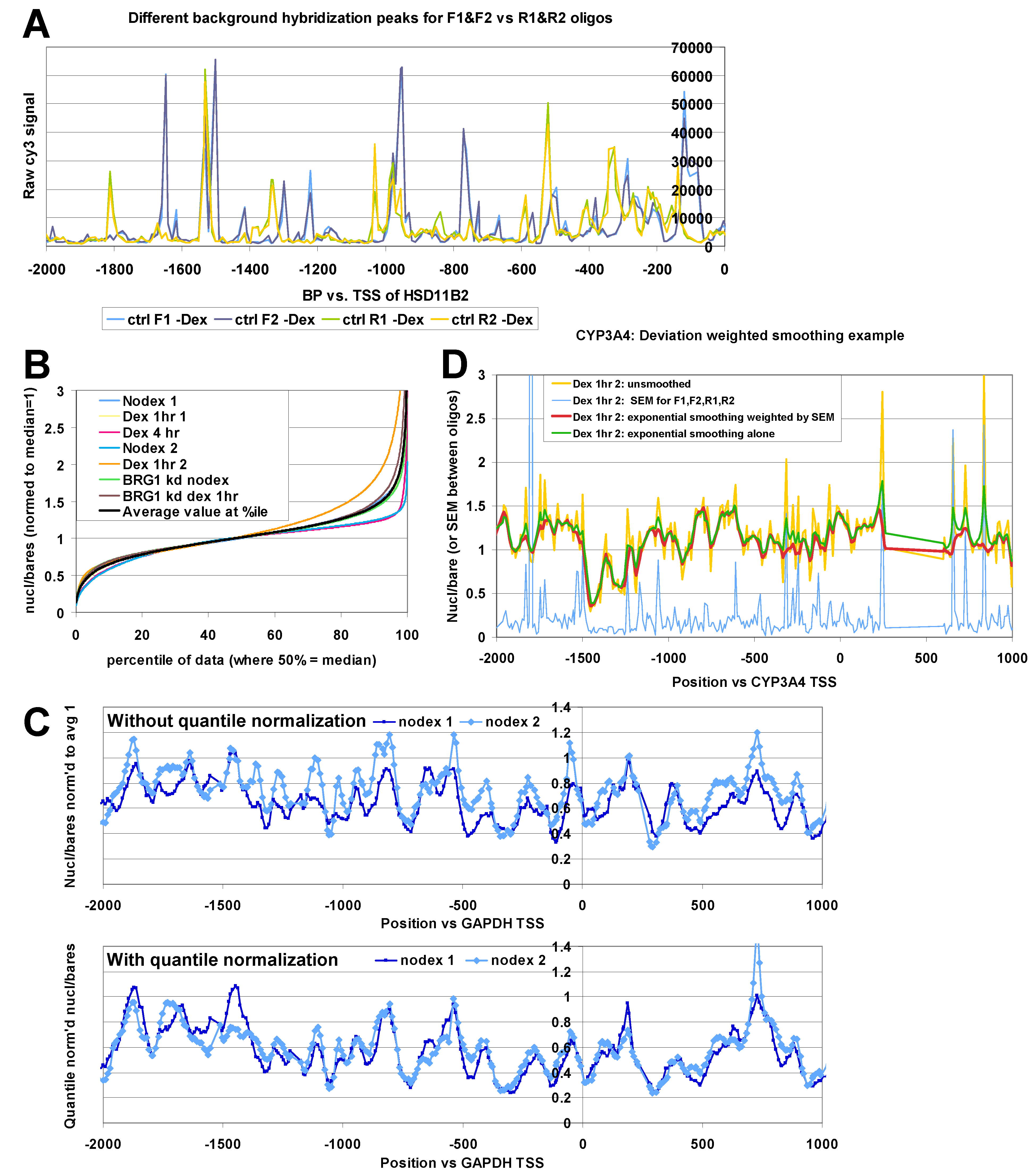

Supplement: Figure S2 — Analytical methods for nucleosome mapping by microarray. (A) Example of F1, F2, R1 and R2 raw data, showing raw cy3 signal from MNase digested bare DNA at HSD11B2. Note how F1 and F2 track together, but often differ from R1 and R2. (B) Curves showing median-normalized nucleosome/bare signal versus percentile in dataset for each UL3 cell experiment. The average curve (black line) was used as the standard for quantile normalization. (C) Comparison of simple median normalization (top) versus quantile normalization (bottom) for the two independent –Dex samples at the GAPDH promoter. In both cases the data was smoothed as described in (D). (D) Example of deviation weighted exponential smoothing. Note how high peaks in the unsmoothed quantile-normalized data (brown line) correspond to regions where the standard error of the median between F1, F2, R1 and R2 for that position are high. Making use of this information in the smoothing function (red line) removes noise peaks that are not effectively removed using normal exponential smoothing (dotted orange line). (TIF) [file pone.0023490.s003.tif]

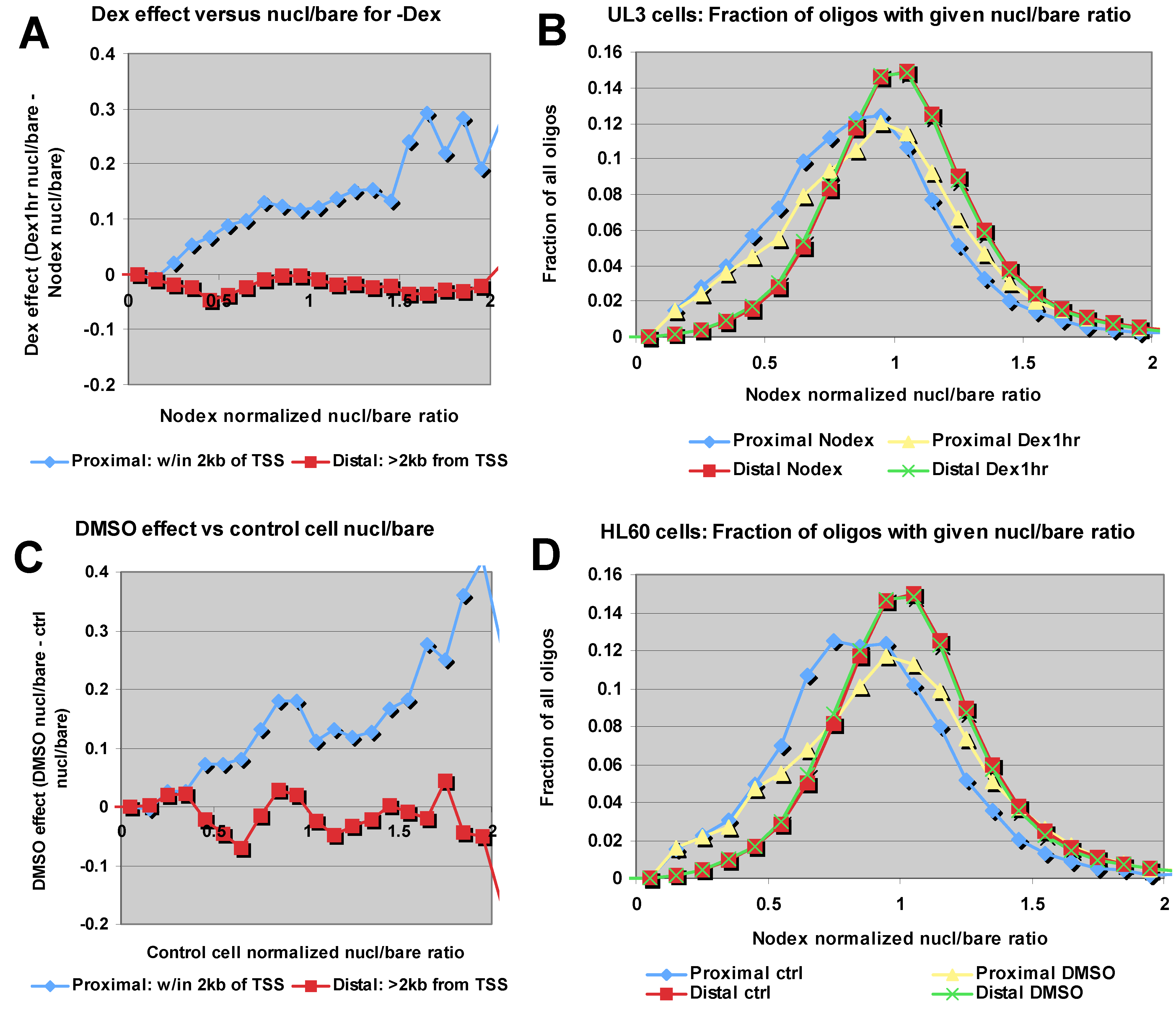

Supplement: Figure S3 — Increased nucleosome occupancy only occurs near TSSes and is not due to hybridization bias. We wished to know whether the increase of nucleosome occupancy surrounding transcription start sites (TSSes) with 1hr Dex treatment of UL3s and with DMSO treatment of HL60s was specific for promoter regions or was also true for other regions of low nucleosome density. In A & C, we tested for this type of systematic bias by calculating the “Dex effect” for each oligo on the array, where Dex effect = (normalized nucl/bare Dex1hr) -(normalized nucl/bare Nodex). Similarly, for HL60 cells, we calculated the DMSO effect: (nucl/bare +DMSO) – (nucl/bare control). We then split the data into two groups, “proximal” for all oligos w/in 2kb of a TSS and “distal” for all oligos more than 2kb away from a TSS. For each nucl/bare ratio under the control condition (-Dex or –DMSO) on the x axis, we then plotted the average Dex or DMSO effect on the y axis. If, for example, there was a hybridization artifact on our +DMSO array that gave an aberrantly high signal at all low-occupancy regions, this would result in a high DMSO effect at nucl/bare ratios less than 1 for both proximal and distal oligos. Instead, increased nucleosome occupancy +Dex1hr or +DMSO was only seen for proximal/promoter regions (blue lines). Furthermore, this was true for oligos showing both low and high occupancy under control conditions (indicating increased occupancy both in troughs as well as at nucleosomal peaks). In B & D, we plotted the fraction of all oligos showing any given nucl/bare ratio (y) versus nucl/bare ratio (x), comparing proximal and distal groups for each treatment condition. Note how the treatment (+DMSO or +Dex 1hr) and control (-DMSO or –Dex) curves for the distal oligos are precisely superimposable (compare red and green curves), indicating no systematic effect of treatment on the distribution of nucleosome occupancies in these regions. By contrast, for proximal oligos, the no treatment curves (blue) [file pone.0023490.s004.tif]

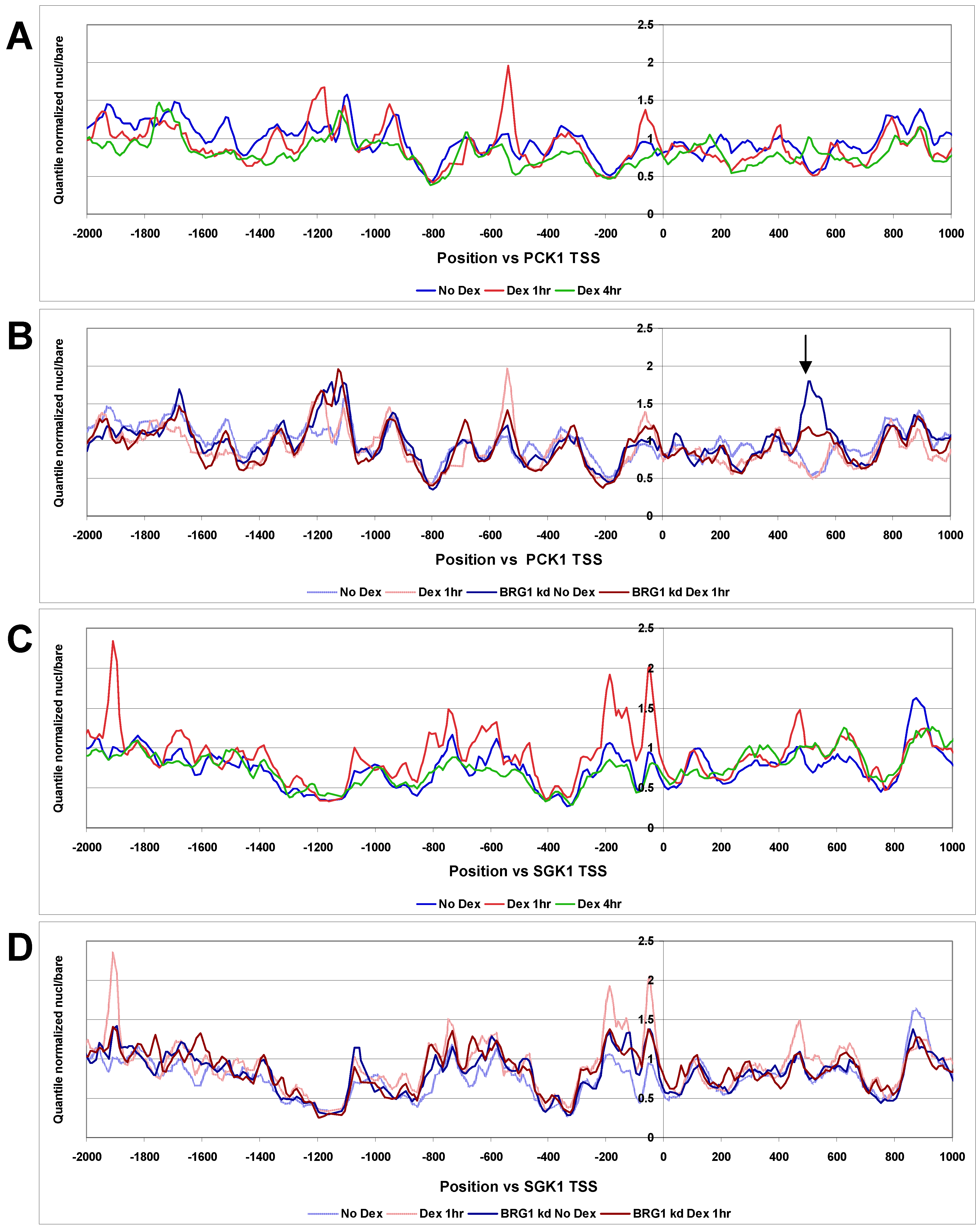

Supplement: Figure S4 — GR activated genes: PCK1 & SGK1 . Promoter nucleosome density of the GR activated genes PCK1/PEPCK (A) & (B), and SGK1 (C) & (D). Figs. S4, S5, S6, S7, S8, S9, S10, S11, S12, S13, S14 show plots of quantile normalized nucleosome/bare ratios for all GR-regulated genes for which densely tiled oligos covered more than 60% of the -2000 to +1000 promoter region (all but SRGN) and most other genes on the array (all but CCNB2, UGT1A6 & UGT1A8). A ratio of 1.0 corresponds to ∼50% nucleosome occupancy and a ratio of 2.1 corresponds to ∼100% nucleosome occupancy (see Fig. 2 legend, or Additional Methods for details). (A) & (C) show -Dex, +Dex 1hr and +Dex 4hr. (B) & (D) show -Dex & +Dex 1hr from control cells (dotted lines) or BRG1 knock down cells (solid lines). The arrow in (B) highlights a BRG1 dependent effect that differs from both + and - Dex control cells. (TIF) [file pone.0023490.s005.tif]

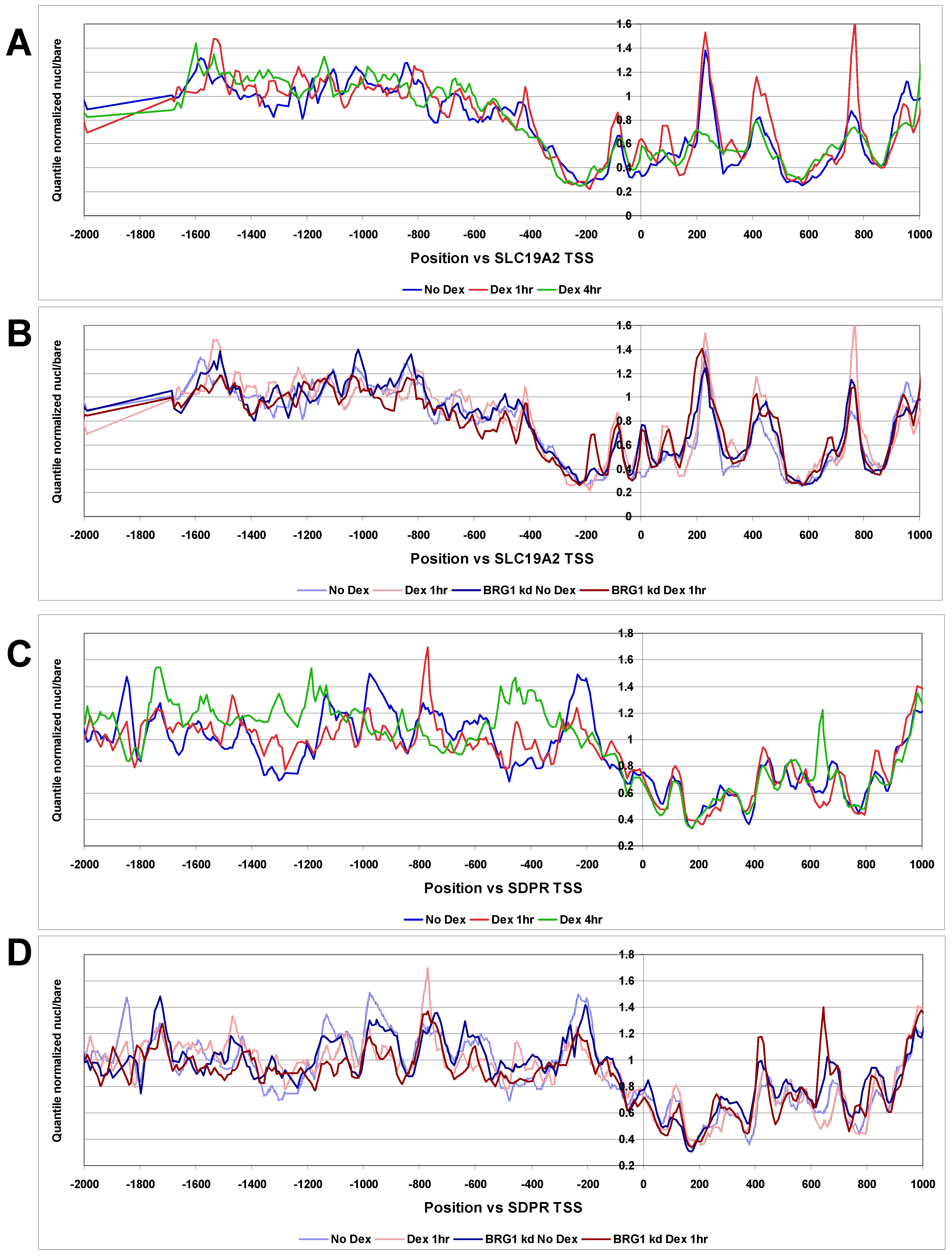

Supplement: Figure S5 — GR activated genes: SLC19A2 & SDPR . Promoter nucleosome density of the GR activated genes SLC19A2 (A) & (B), and SDPR (C) & (D), as described in Figure S4. (A) & (C) show -Dex, +Dex 1hr and +Dex 4hr. (B) & (D) show -Dex & +Dex 1hr from control cells (dotted lines) or BRG1 knock down cells (solid lines). (TIF) [file pone.0023490.s006.tif]

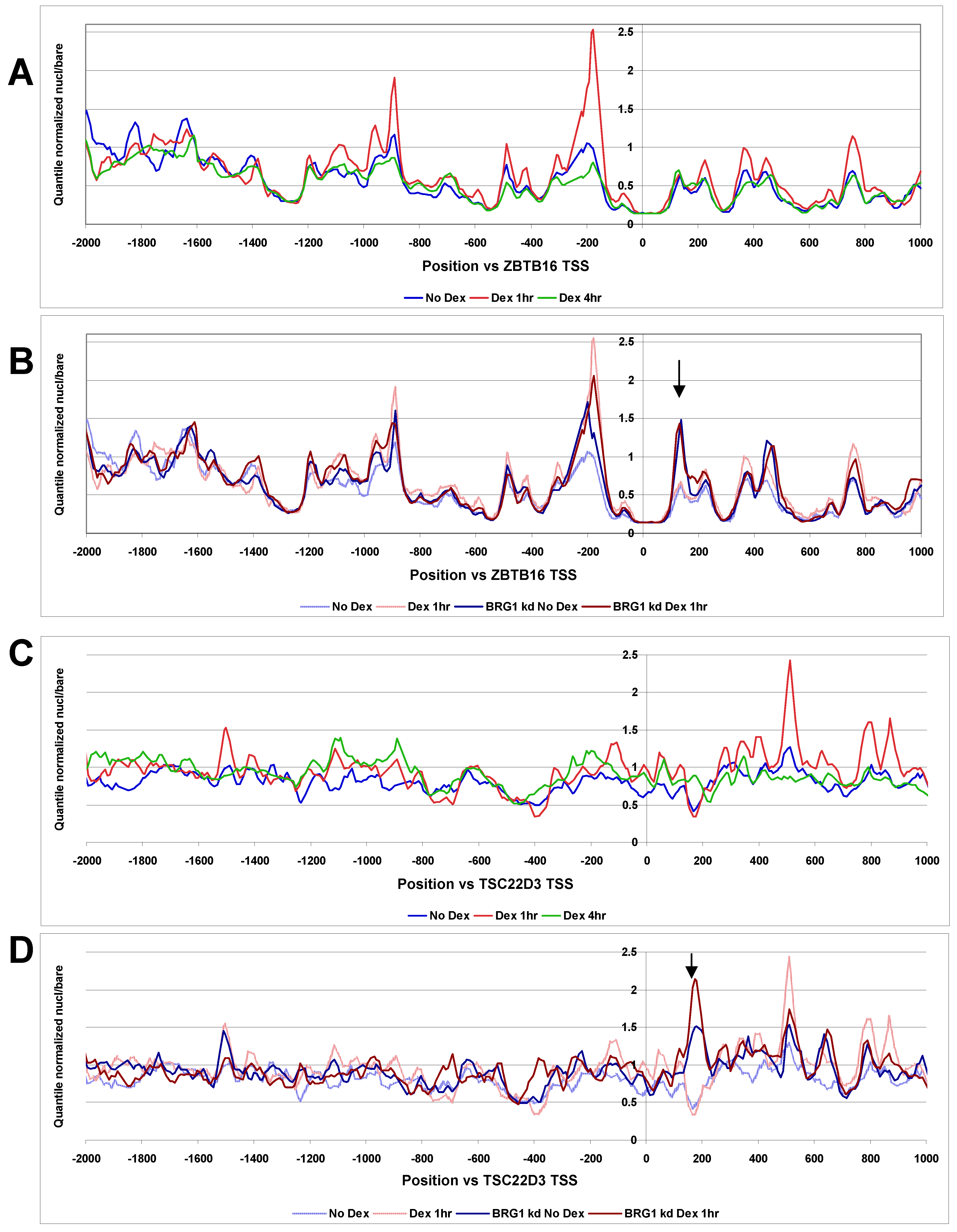

Supplement: Figure S6 — GR activated genes: ZBTB16 & TSC22D3 . Promoter nucleosome density of the GR activated genes ZBTB16/PLZF (A) & (B), and GILZ/TSC22D3 (C) & (D), as described in Figure S4. (A) & (C) show -Dex, +Dex 1hr and +Dex 4hr. (B) & (D) show -Dex & +Dex 1hr from control cells (dotted lines) or BRG1 knock down cells (solid lines). The arrows in (B) & (D) highlight BRG1 dependent effects that differ from both + and - Dex control cells. (TIF) [file pone.0023490.s007.tif]

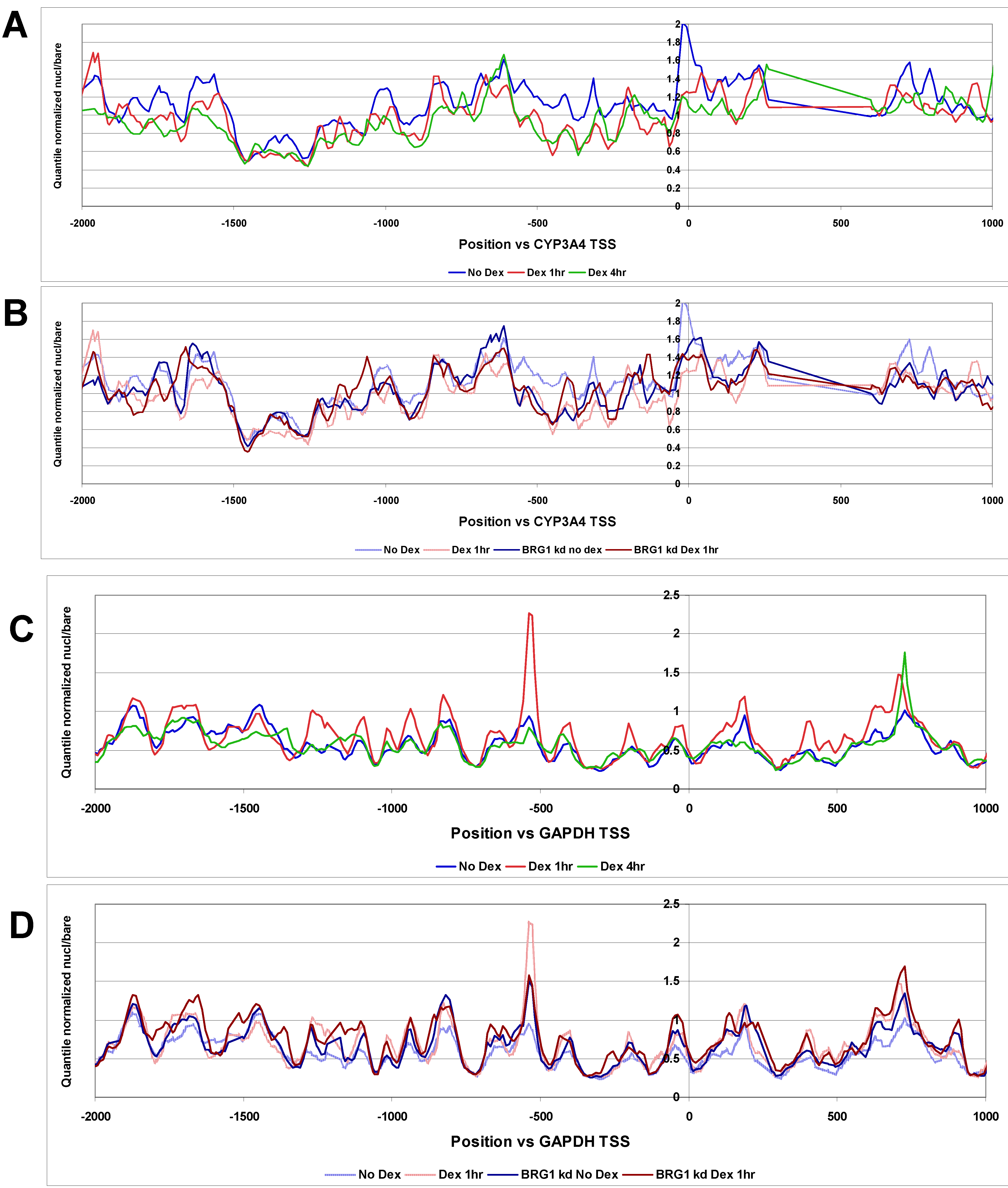

Supplement: Figure S7 — GR activated gene CYP3A4 and control gene GAPDH . Promoter nucleosome density for the GR activated gene CYP3A4 (A) & (B) and the GR- and hSWI/SNF-unregulated gene GAPDH (C) & (D), as described in Figure S4. (A) & (C) show -Dex, +Dex 1hr and +Dex 4hr. (B) & (D) show -Dex & +Dex 1hr from control cells (dotted lines) or BRG1 knock down cells (solid lines). (TIF) [file pone.0023490.s008.tif]

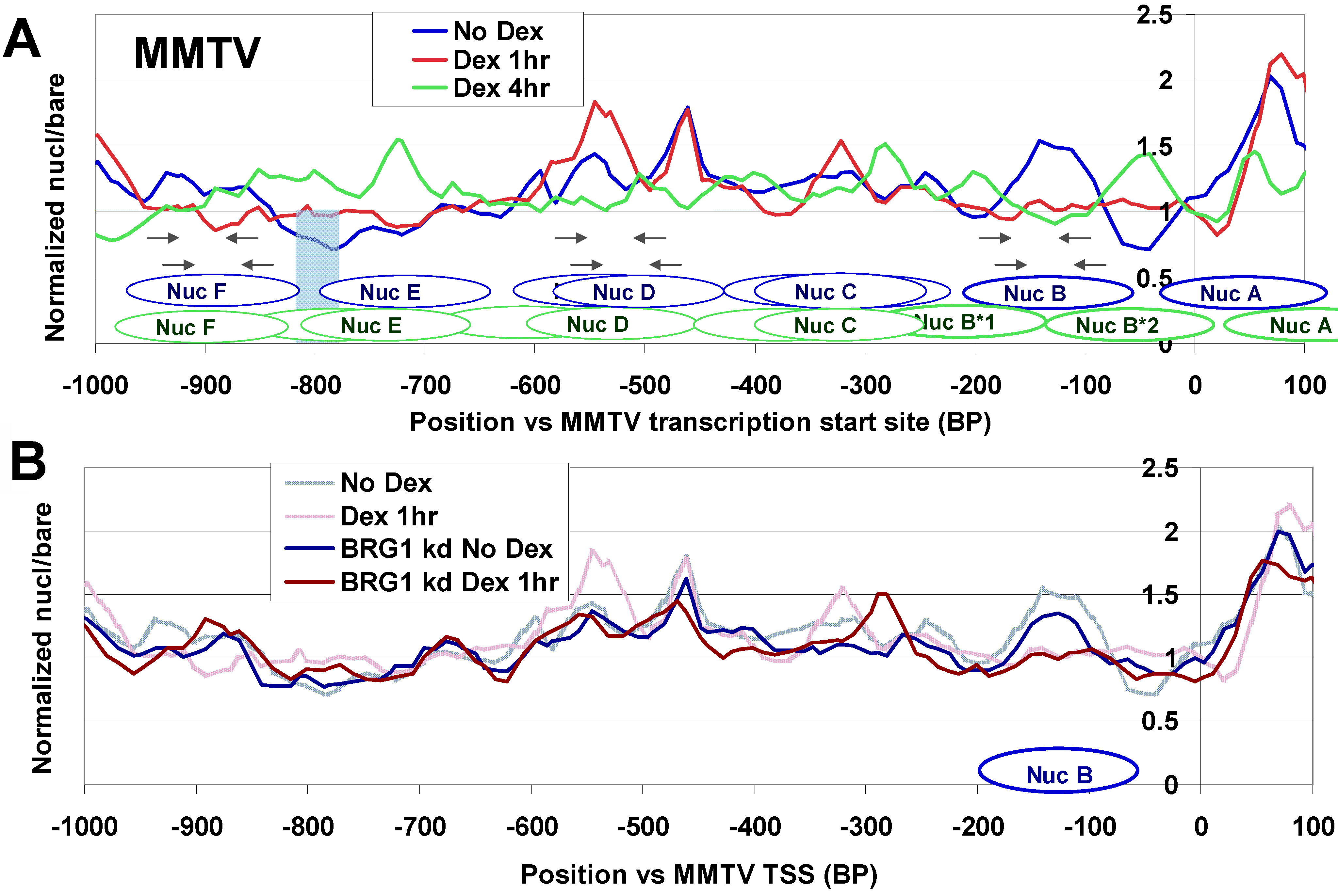

Supplement: Figure S8 — GR-activated MMTV promoter. (A) Same as Fig. 2A (reproduced here for comparison). Note that the relative weakness of Nuc C, and the split nature of Nucs D through F, is consistent with microarray mapping results for an MMTV construct integrated into another cell line (MDA-kb2 cells, Dennis et al. (2007) Genome Res 17:928-39), and may also be consistent with indirect end-labeling results which sometimes showed multiple local peaks of nuclease sensitivity in the gaps between Nucs C through Nucs E (e.g. Trotter & Archer (2004) Mol Cell Biol 24:3347-58, Truss et al. (1995) EMBO J 14:1737-51, and Richard-Foy & Hager (1987) EMBO J 6:2321-28). Importantly, indirect end-labeling identifies positioned nucleosomes by the appearance of relatively strong MNase digestion sites flanking ∼146 bp regions of relative protection. These conditions can occur regardless of the fraction of gene copies that bear a positioned nucleosome. Thus, indirect end labeling is expected to provide information about nucleosome edges but will not provide accurate information about relative nucleosomal occupancy. For instance, while the c-myc promoter Nucleosomes 7 and 8 (identified by indirect end labeling) did appear as discrete nucleosome position peaks in our analysis, the relative weakness of these peaks suggests that these positioned nucleosomes are present on only a subset of gene copies (Fig. 2C). (B) Effects of BRG1 knock down on MMTV promoter chromatin. Dotted lines show nucleosome density maps -Dex & +Dex 1hr from control cells (from A) and solid lines show –Dex and +Dex 1hr results from BRG1 knock down cells. Note that NucB decreases +Dex 1hr in the BRG1 knock down as well as control cells (an effect that was confirmed by MNase footprint PCR on mononucleosomal fragments, which showed that 1hr dex treatment of BRG1 knock down cells caused Nuc B occupancy to decrease to 57 +/- 16% of –Dex levels, similar to the results from control cells in Fig. 2B). This was somewhat unexpected, given that p [file pone.0023490.s009.tif]

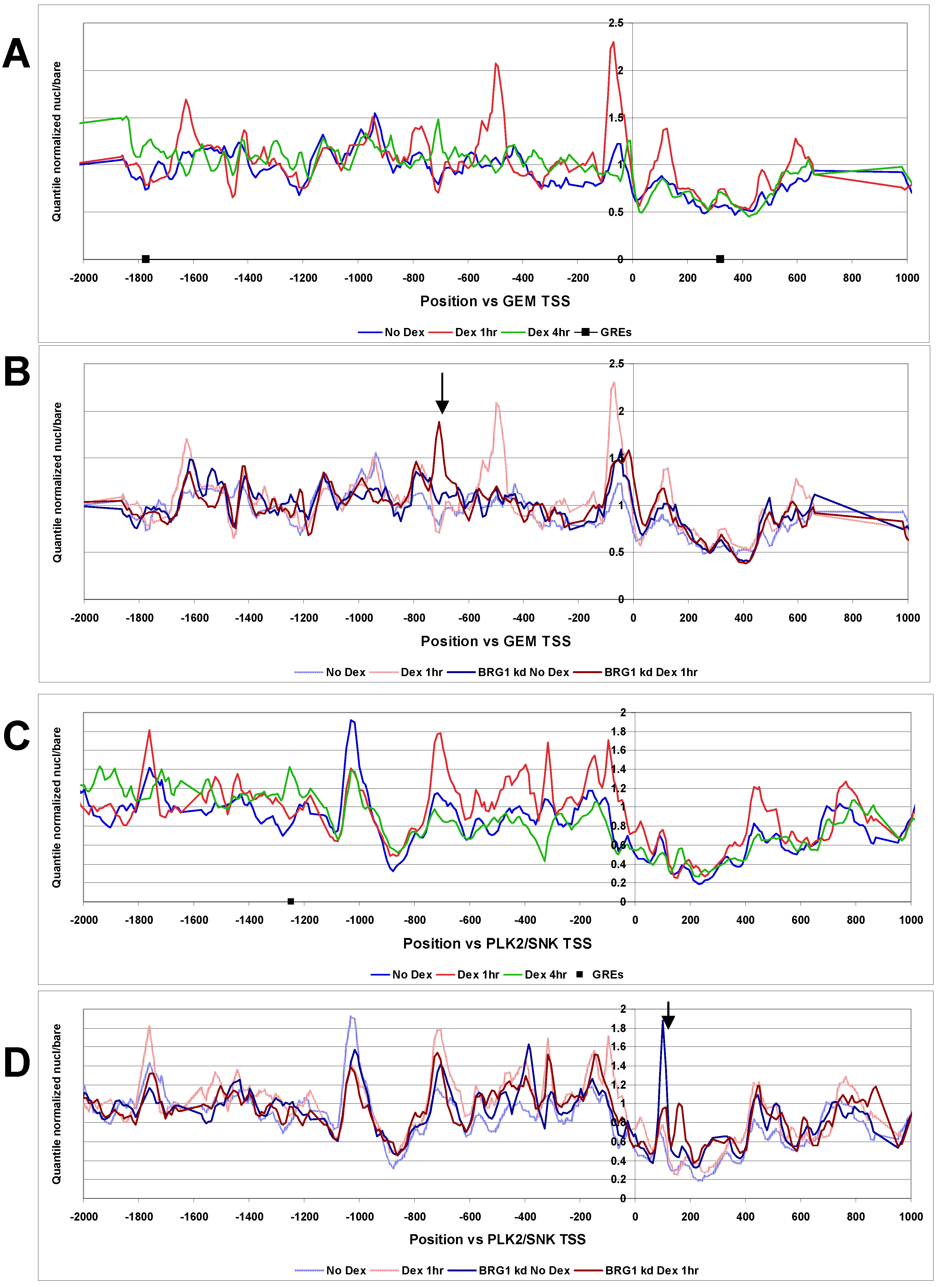

Supplement: Figure S9 — GR repressed genes: GEM & PLK2 . Promoter nucleosome density of the GR repressed genes GEM (A) & (B), and PLK2/SNK (C) & (D), as described in Figure S4. (A) & (C) show -Dex, +Dex 1hr and +Dex 4hr. (B) & (D) show -Dex & +Dex 1hr from control cells (dotted lines) or BRG1 knock down cells (solid lines). The arrows in (B) & (D) highlight BRG1 dependent effects that differ from both + and - Dex control cells. In (A) and (C), previously-mapped GR binding sites are indicated by squares. (TIF) [file pone.0023490.s010.tif]

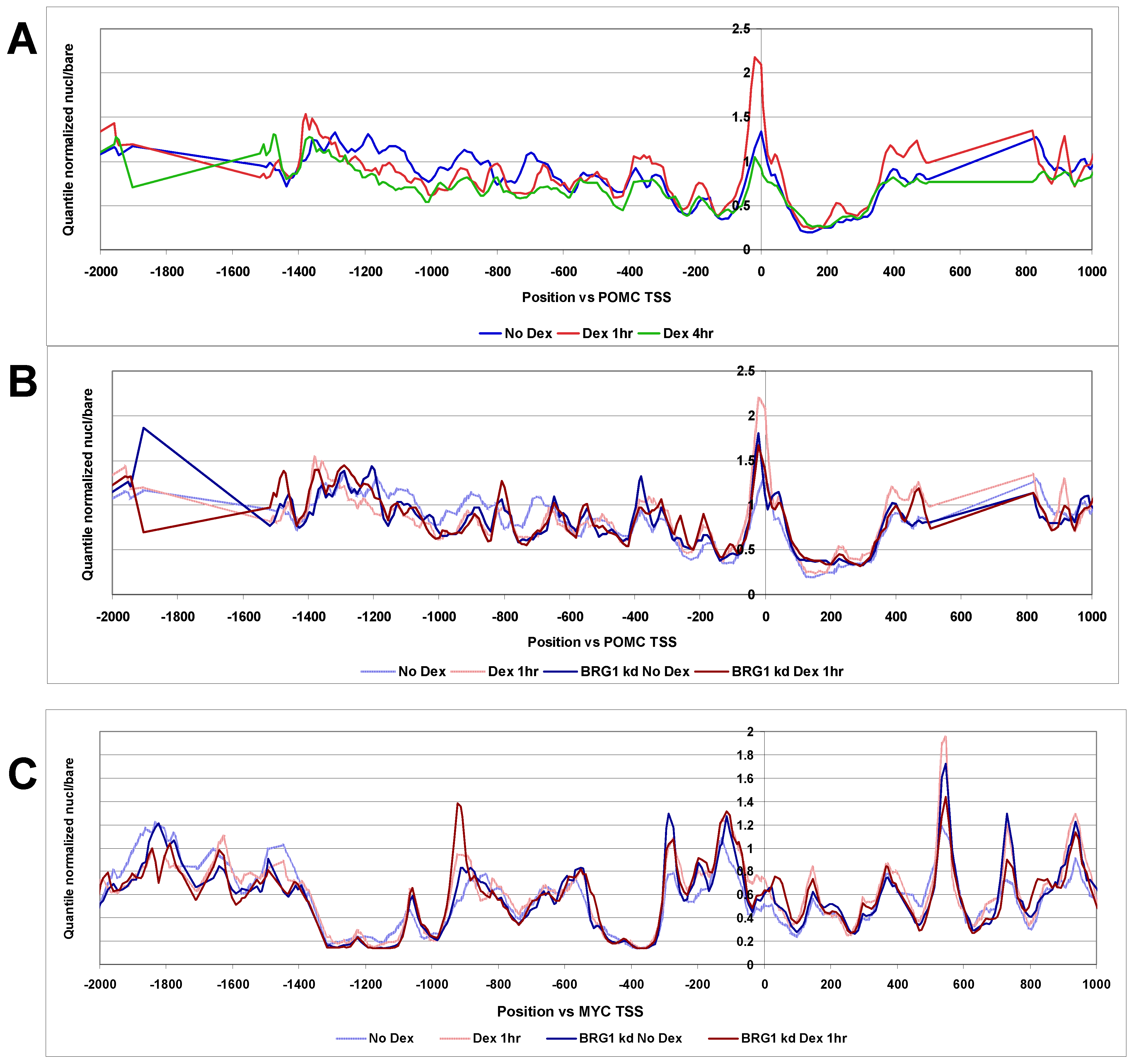

Supplement: Figure S10 — GR repressed genes: POMC & MYC . Promoter nucleosome density of the GR repressed genes POMC (A) & (B), and MYC (C), as described in Figure S4. (A) shows -Dex, +Dex 1hr and +Dex 4hr for POMC. For +Dex 4hr results on myc, see Fig. 2C. (B) & (C) show -Dex & +Dex 1hr from control cells (dotted lines) or BRG1 knock down cells (solid lines). (TIF) [file pone.0023490.s011.tif]

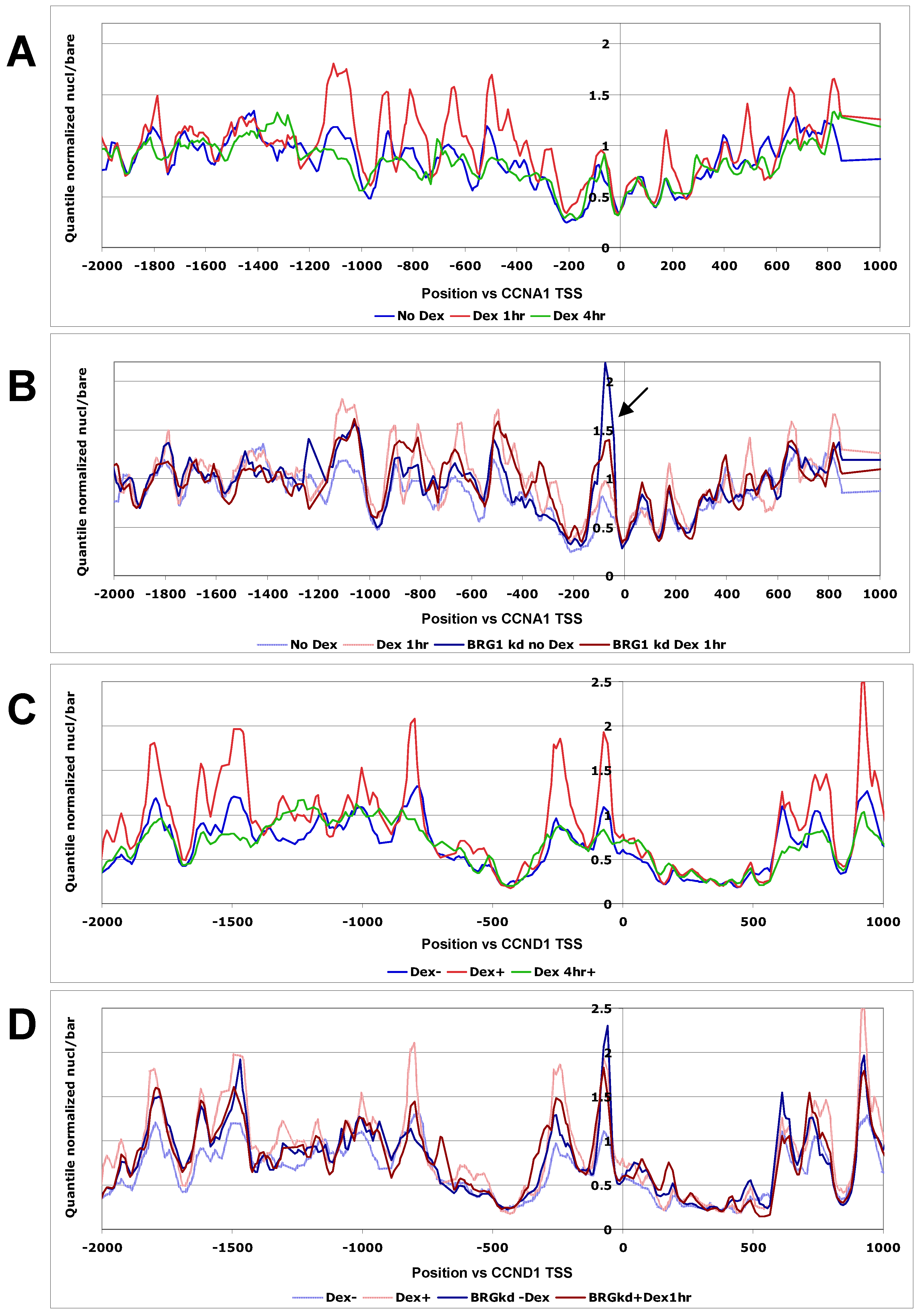

Supplement: Figure S11 — Non-GR regulated cell cycle control genes: CCNA1 & CCND1 . Promoter nucleosome density of the GR-unregulated genes CCNA1/cyclin A1 (A) & (B), and CCND1/cyclin D1 (C) & (D), as described in Figure S4. (A) & (C) show -Dex, +Dex 1hr and +Dex 4hr. (B) & (D) show -Dex & +Dex 1hr from control cells (dotted lines) or BRG1 knock down cells (solid lines). The arrow in (B) highlights a BRG1 dependent effect that differs from both + and - Dex control cells. (TIF) [file pone.0023490.s012.tif]

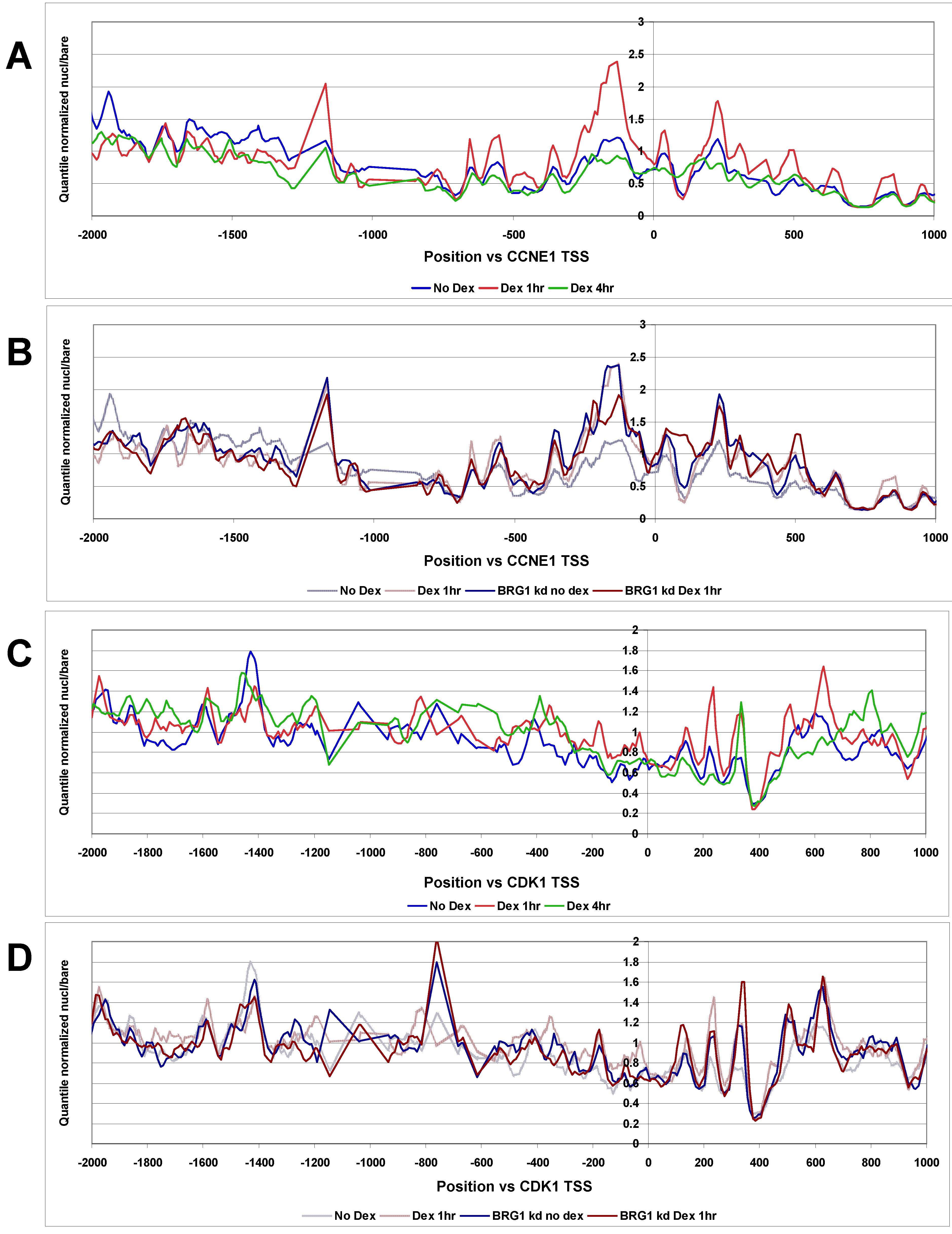

Supplement: Figure S12 — Non-GR regulated cell cycle control genes: CCNE1 & CDK1 . Promoter nucleosome density of the GR unregulated genes CCNE1/cyclin E1 (A) & (B), and CDK1/CDC2 (C) & (D), as described in Figure S4. (A) & (C) show -Dex, +Dex 1hr and +Dex 4hr. (B) & (D) show -Dex & +Dex 1hr from control cells (dotted lines) or BRG1 knock down cells (solid lines). (TIF) [file pone.0023490.s013.tif]

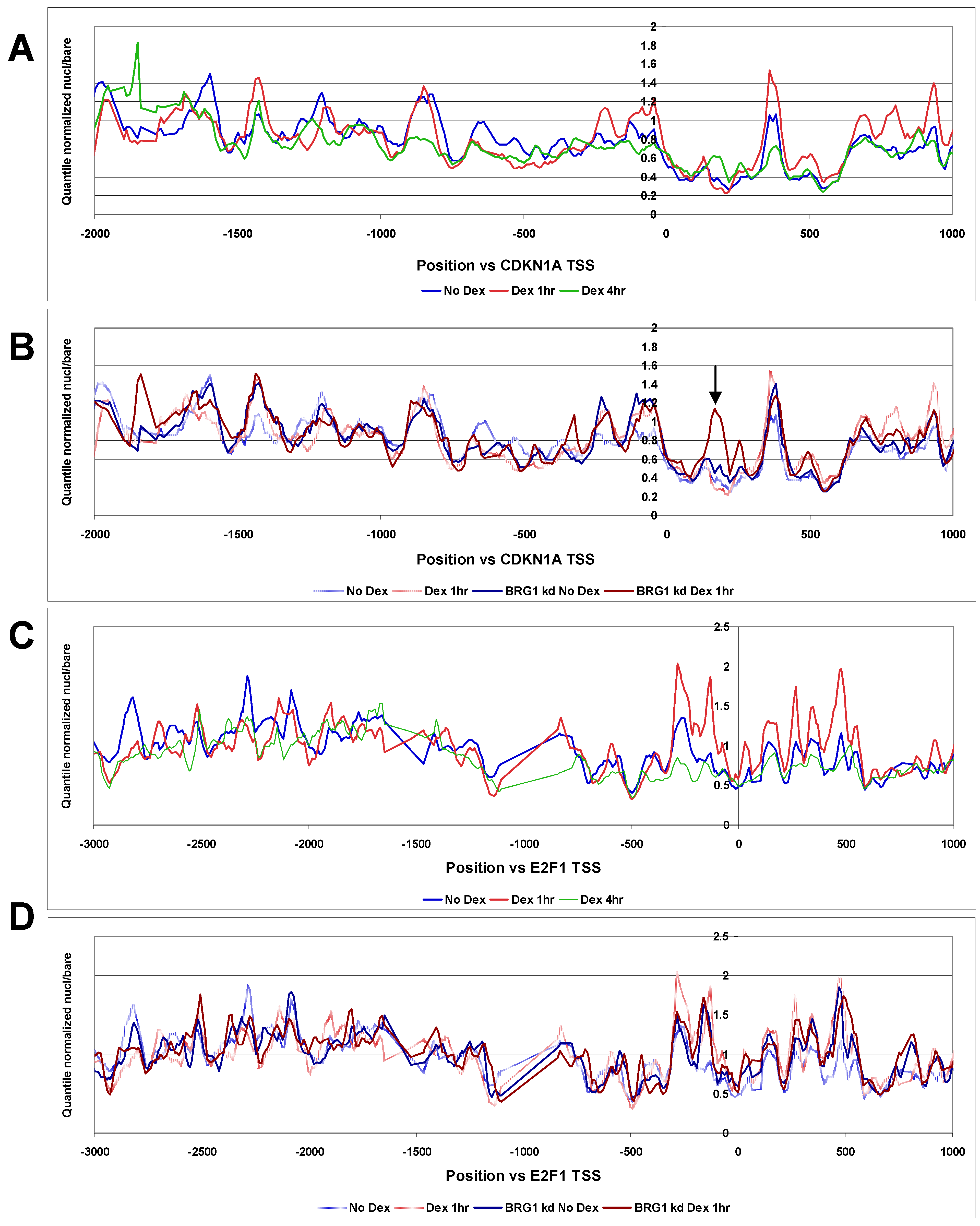

Supplement: Figure S13 — Non-GR regulated cell cycle control genes: CDKN1A & E2F1 . Promoter nucleosome density of the GR unregulated genes CDKN1A/p21 (A) & (B), and E2F1 (C) & (D), as described in Figure S4. (A) & (C) show -Dex, +Dex 1hr and +Dex 4hr. (B) & (D) show -Dex & +Dex 1hr from control cells (dotted lines) or BRG1 knock down cells (solid lines). The arrow in (B) highlights a BRG1 dependent effect that differs from both + and - Dex control cells. (TIF) [file pone.0023490.s014.tif]

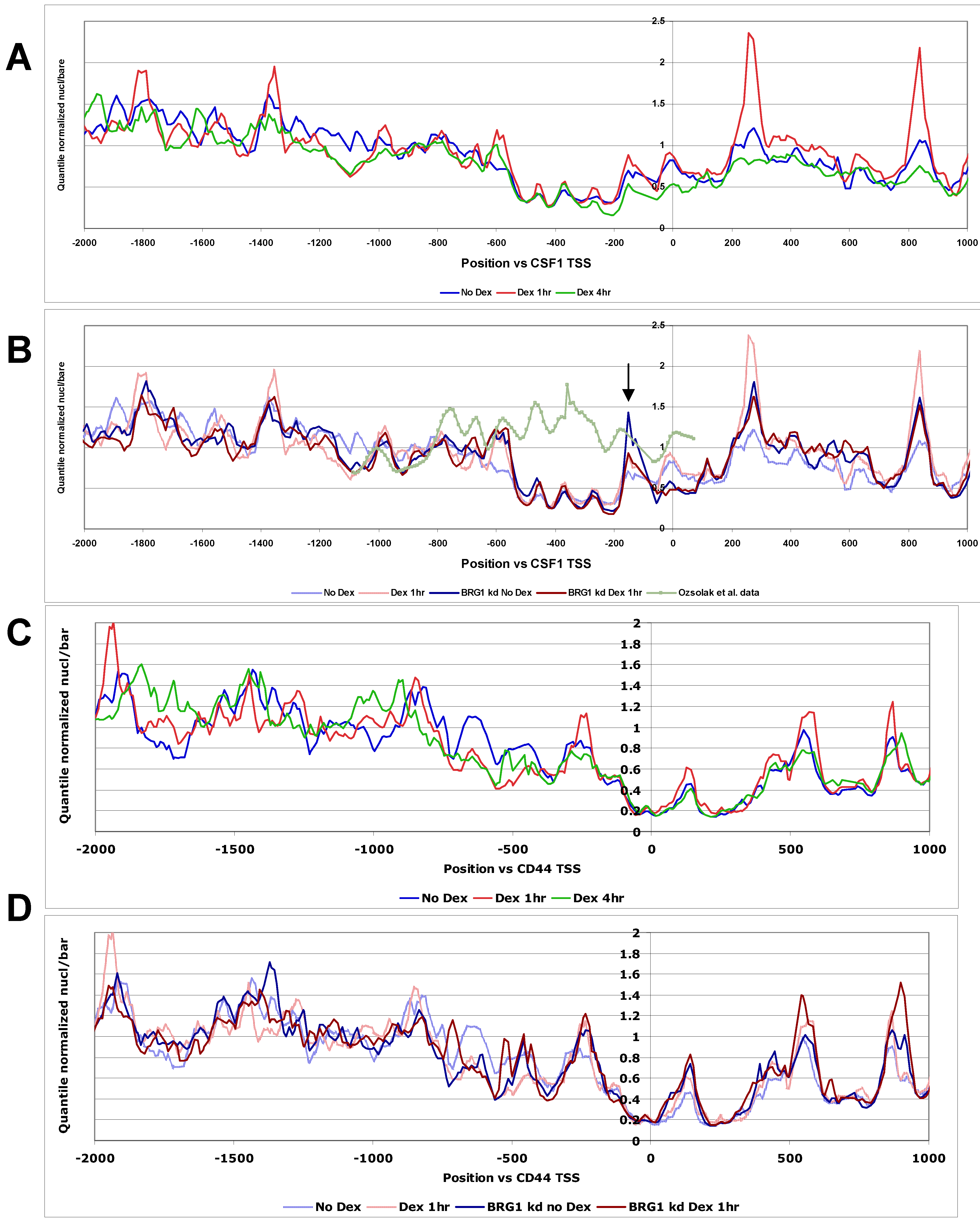

Supplement: Figure S14 — Non-GR regulated, hSWI/SNF-regulated genes: CSF1 & CD44 . Promoter nucleosome density of the GR-independent, hSWI/SNF activated genes CSF1 (A) & (B), and CD44 (C) & (D), as described in Figure S4. (A) & (C) show -Dex, +Dex 1hr and +Dex 4hr. (B) & (D) show -Dex & +Dex 1hr from control cells (dotted lines) or BRG1 knock down cells (solid lines). The arrow in (B) highlights a BRG1 dependent effect that differs from both + and - Dex control cells. The green line in (B) shows data from Ozsolak et al. 2007, Nat Biotechnol 25:244-8 (GEO accession # GSE6385). (TIF) [file pone.0023490.s015.tif]

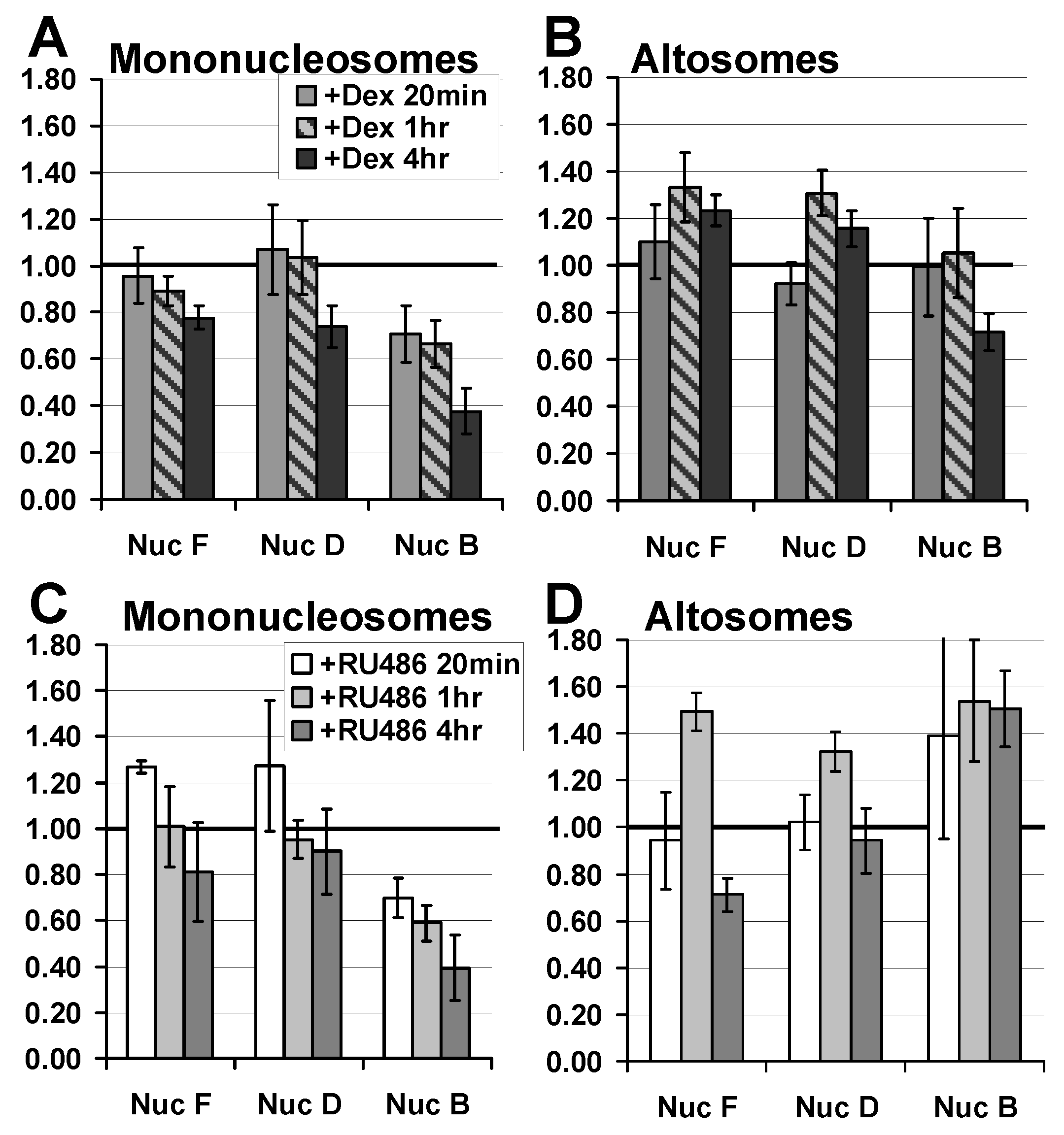

Supplement: Figure S15 — Loss of Nuc B mononucleosomes is not due to altered dinucleosome formation by hSWI/SNF. (A) Mononucleosome levels at MMTV NucF, NucD and NucB at the indicated times, as measured by MNase footprint PCR (repeated from Fig. 2B, to facilitate comparison). (B) Altered dinucleosomes formed by hSWI/SNF (altosomes) have an unusual ∼200 bp MNase footprint size, which allowed us to assay altosome levels on MMTV by subjecting ∼200 bp MNase-resistant chromatin fragments to MNase footprint PCR. The results showed that altosome levels at Nuc B did not rise significantly after 20 mins or 1 hour of dex treatment, and actually decreased at 4 hrs, arguing against the hypothesis that NucB is converted into altosomes. Interestingly, an increase in altosome formation was observed at Nuc F and D at 1 hr., which moved towards baseline levels after 4 hr. Because the footprint size of altosomes cannot arise from any arrangement of normal nucleosomes, and because no other remodeling complex is known to form altosomes, this observation suggests that hSWI/SNF affects nucleosomes up to ∼1 kb beyond its expected sites of recruitment (the GRE elements located within Nuc B). A return of altosomes to baseline levels at 4 hr is consistent with altosomes' innate tendency to revert to normal nucleosomes. (C) Prior studies indicate that, the partial agonist RU486 induces GR binding to MMTV and recruitment of hSWI/SNF, but does not support the recruitment of other coactivators, including pCIP, SRC1, and p300 (Fryer CJ et al. (2000) J Biol Chem, 275:17771-7), allowing the examination of hSWI/SNF effects independent of other GR coactivators. Consistent with these studies, we find that RU486 decreases the amount of MMTV reporter induction compared to dexamethasone (to 76 +/- 6 fold after 24hrs, as compared to 513 +/- 27 fold with Dex). When mononucleosome positions were analyzed by MNase footprint PCR for UL3 cells treated with RU486, we found that reduction of Nuc B occupancy was apparent after 20 min. an [file pone.0023490.s016.tif]

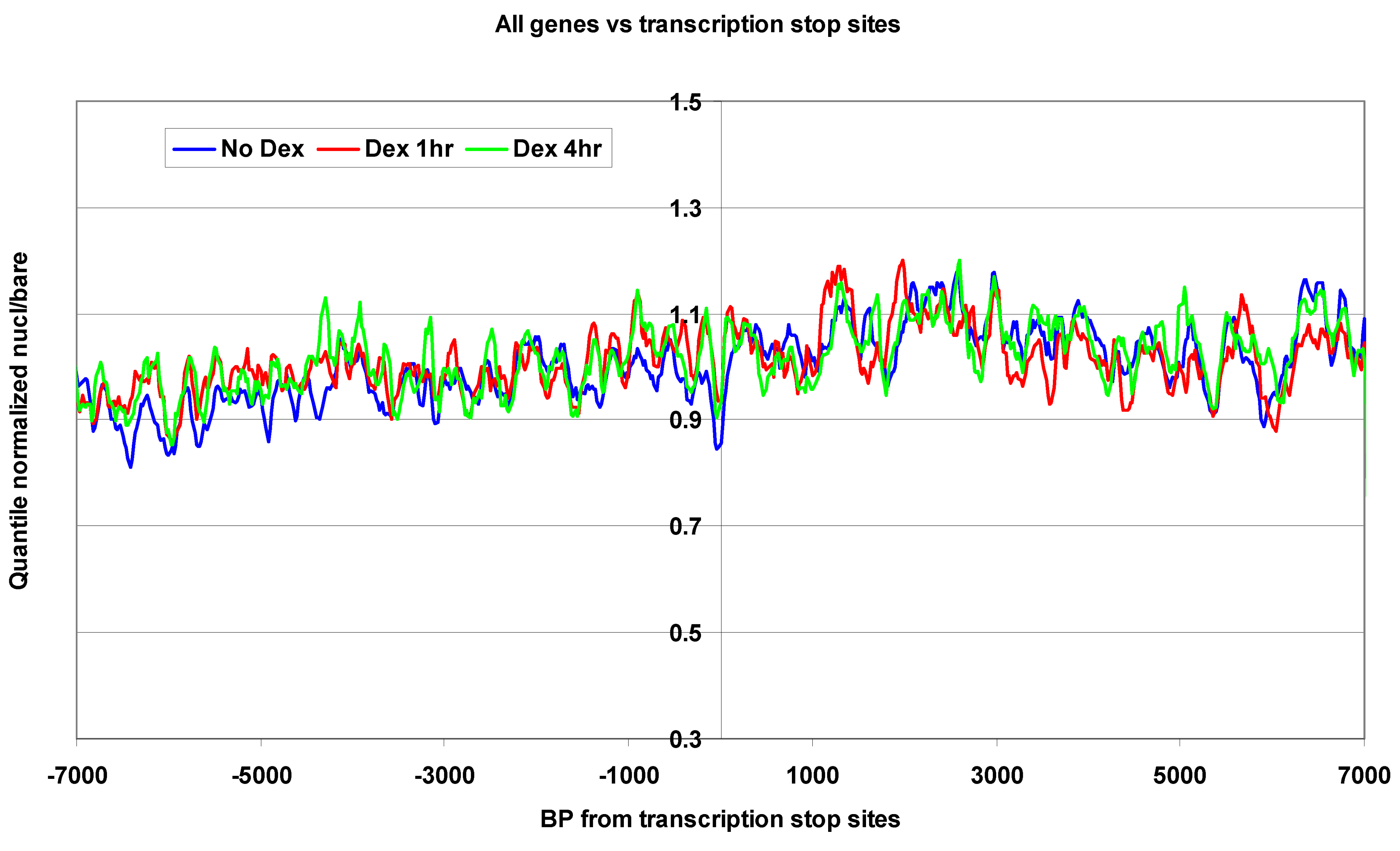

Supplement: Figure S16 — Dex treatment does not significantly alter nucleosome density at transcription termination sites. As for Fig. 5A, but looking at average nucleosome density relative to transcription termination sites (ends of transcribed regions), for all endogenous genes for which nucleosome positions were mapped at least 5 kb downstream of termination sites. (TIF) [file pone.0023490.s017.tif]

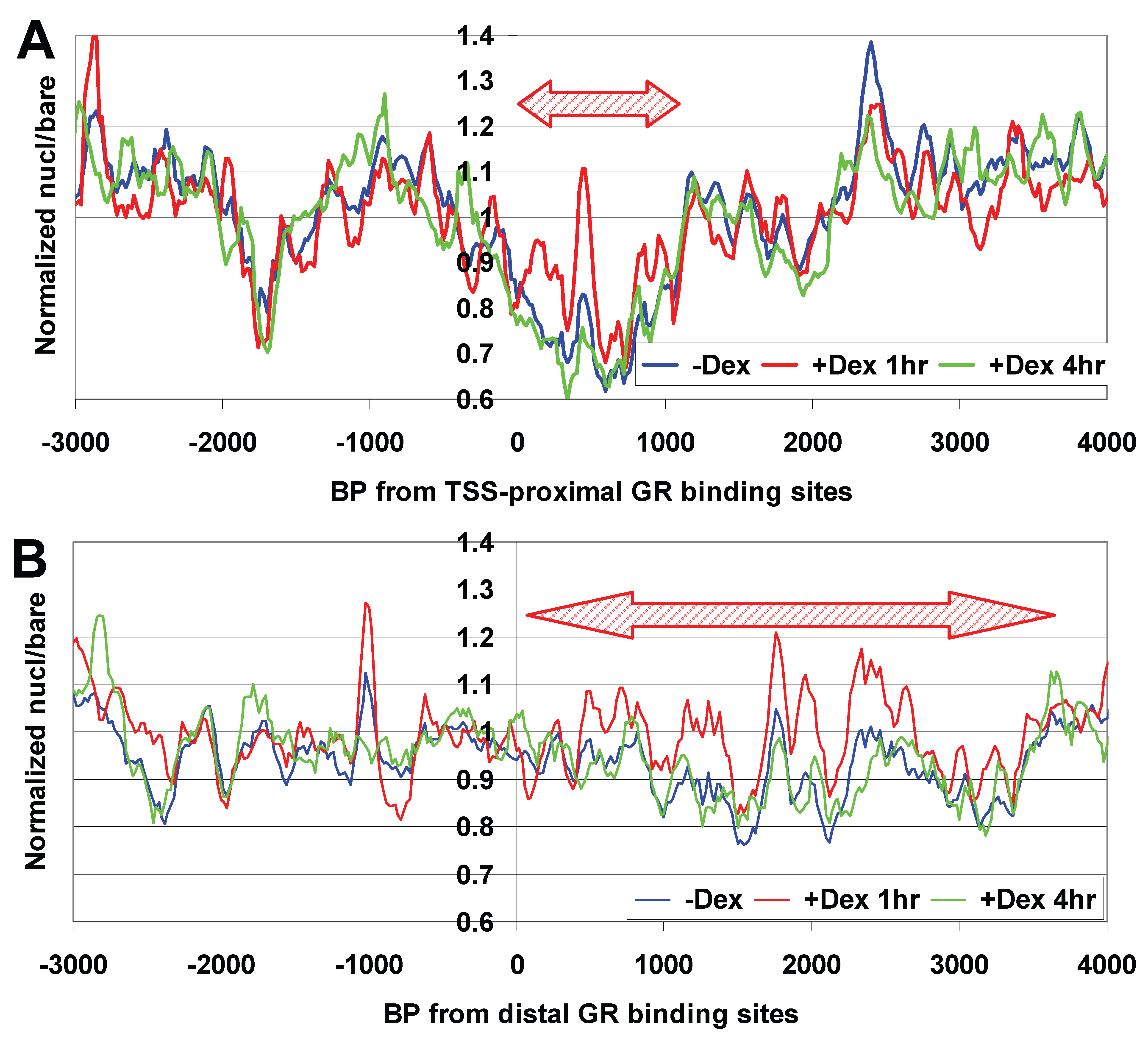

Supplement: Figure S17 — Remodeled chromatin extends further downstream of distal GR binding sites. (A) Average nucleosome occupancy mapped relative to GR binding sites that map to within 500 bp upstream of the TSS of their regulated genes (locations relative to TSSes: SDPR ∼-50, SRGN ∼-300, SLC19A2 ∼-150, GEM ∼-320, and POMC ∼-400). (B) Average nucleosome occupancy relative to GR binding sites greater than 1kb upstream of regulated TSSes (locations relative to TSSes: HSD11B2 ∼1500, SDPR ∼19800, SGK1 ∼-1290, TSC22D3∼1700, and GEM ∼1770, PLK2 ∼1250 & ∼ -2700). See Table S1 for details. (TIF) [file pone.0023490.s018.tif]

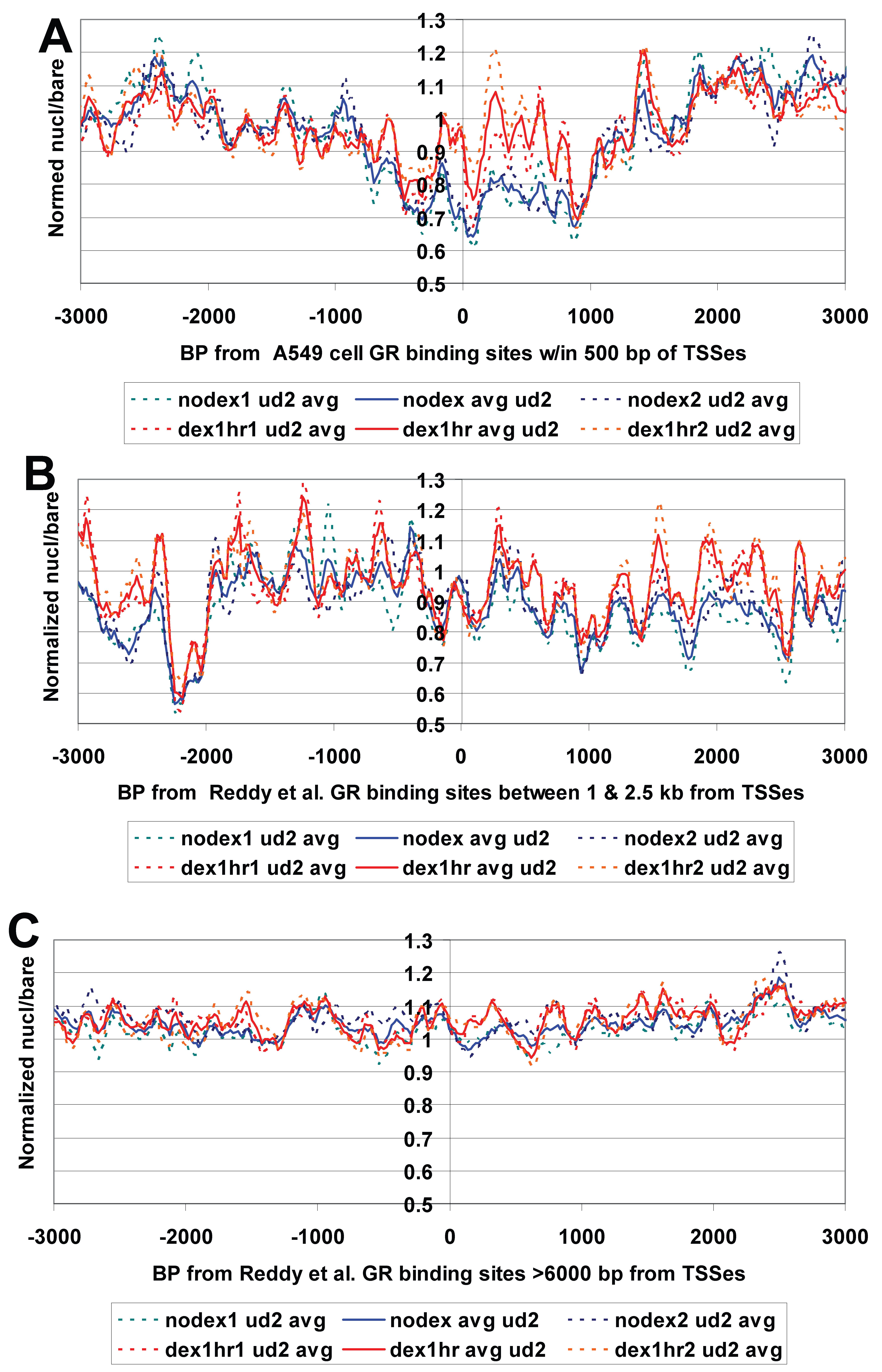

Supplement: Figure S18 — Changes in nucleosome occupancy relative to GR binding sites in human A549 lung cells. As for Fig. S17, but looking at average nucleosome density relative to subsets of GR binding sites identified in human A549 lung carcinoma cells (Reddy et al. 2009, Genome Res 19:2163-71). (A) all six GR binding sites within 500 bp of TSSes. (B) all five GR binding sites between 1 and 2.5 kb of TSSes. (C) all 37 GR binding sites covered by our array and at least 6kb from TSSes. Dotted lines show the results from repeat samples. Solid lines show the average result. (TIF) [file pone.0023490.s019.tif]

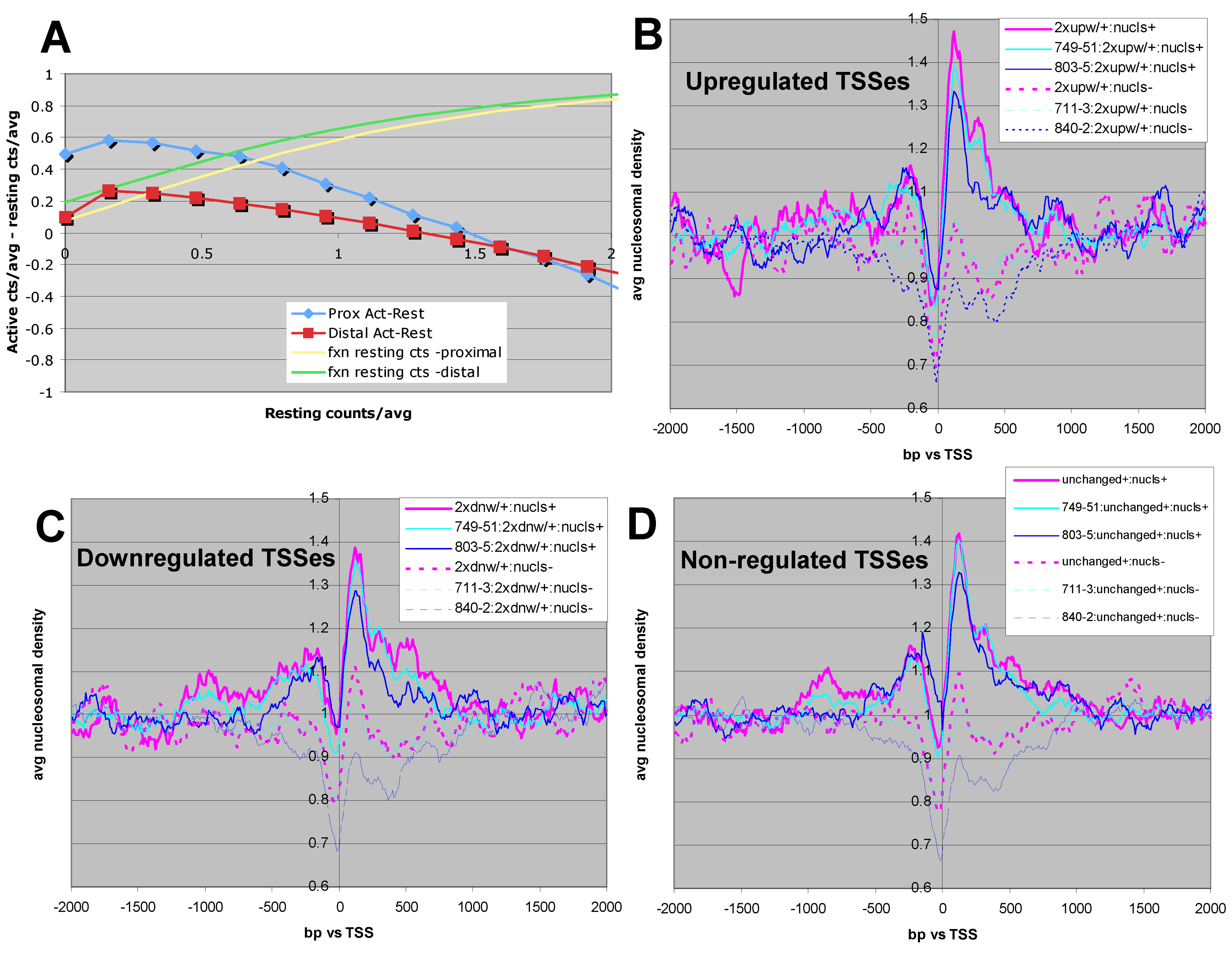

Supplement: Figure S19 — Increased nucleosome occupancy with CD4+ T-cell activation is not due to general bias. A) We examined the nucleosome count data from Schones et al. using a similar metric to that used for our microarray data in Fig. S3. Briefly, we mapped the treatment effect (acttivated_nucl_counts/avg_activated_nucl_counts) – (resting_nucl_counts/avg_resting_nucl_counts) versus normalized nucleosome density from the resting data (resting_nucl_counts/avg_resting_nucl_counts) for all nucleosome count data within 500 bp of TSSes (proximal) or all data more than 500 bp from any known TSS (distal). We found that a strong increase in nucleosome density was only seen at promoters (blue line) and not in distal regions (red line). In contrast to our microarray results, there was a distinct tendency towards a weak positive effect at nucleosome count values less than 1.0 (below average) and an increasing negative effect at values greater than 1.0 (the negative slope of red line). This would be expected based on the discrete count nature of the sequencing data. For example, when inherent sampling variability in sequencing gives a reads/avg_reads value that is higher than the actual value for one sample, a second sample is likely to yield a reads/avg_reads value that is closer to the actual value. This would result in some degree of apparent negative treatment effect at high reads/avg values, and positive effect at low values. Importantly, even though the strong treatment effect at promoters goes away at nucl_count/avg_nucl_count ratios greater than ∼1.5, positions with these read values make up 75-80% of all reads within the proximal (yellow curve) and distal (green curve) regions. Hence increased nucleosome occupancy is specifically seen around TSSes at the ∼75% of locations where nucl/avg_nucl values are below ∼1.5x genomic average. B-D) The Schones et al data consists of more than 50 Illumina sequencing lanes per condition that appear to have been derived from several distinct samples. To [file pone.0023490.s020.tif]
