# Supplementary material for: Multiple Distinct Stimuli Increase Measured Nucleosome Occupancy around Human Promoters
Source: PLoS One. 2011 Aug 11;6(8):e23490. doi: 10.1371/journal.pone.0023490 (PMC3154950; doi:10.1371/journal.pone.0023490)
Supplement: Table S1 — Genes covered by the nucleosome mapping Nimblegen arrays. Blue: genes activated by GR and Dex. Pink: genes repressed by GR and Dex. Grey: cell cycle control genes that are not regulated by GR and Dex in U20S cells. For citations given in the table, see Additional Methods, in Text S1. (DOC) [file pone.0023490.s021.doc]

| **Table S1: Genes covered by the nucleosome mapping Nimblegen arrays** | |
| --- | --- |
| ***MMTV & HSD11B2*** | Both 11*β* hydroxysteroid dehyrdogenase (*HSD11B2*) and MMTV require hSWI/SNF for coactivation via GR and NF1 [11,12]. The array covers the complete sequence of the MMTV-luc vector that was integrated into U2-OS cells to generate the UL3 line. GR binds to the Nuc B region (~-150 bp from TSS) and ~-1500 from the TSS of HSD11B2 [12] |
| ***PCK1, SDPR, SGK1, SLC19A2, TSC22D3, ZBTB16, SRGN & CYP3A4*** | Primary endogenous target genes upregulated by hormone bound GR [11,12,13,14,16]. *ZBTB16/PLZF* requires hSWI/SNF for its activation by dex [17]. Microarray analysis in U2OS cells expressing GR showed induction after 6 hr dex treatment as follows: *PCK1/PEPCK* & *CYP3A4* (1.9x), *TSC22D3/GILZ* (1.3x), *SGK1* &  *ZBTB16* (1.2x) ([18], GEO accession # GSE 11205). A second microarray experiment in U20S cells found three genes showing induction greater than the 2-fold cut-off used after 2 hr dex treatment::  *SGK1* (8x),  *TSC22D3* (3.4x) & *SDPR* (2x) [19]. GR binding sites identified in prior ChIP studies were located, relative to TSSes, at: *SDPR* -49 to -169, *SLC19A2* -135 to -254, *TSC22D3* -2295 to -1705, *SRGN* -252 to -357 [13], *SDPR* ~+12 & -19787, *SGK1* ~-1380, *SLC19A2* ~-136, *TSC22D3* ~-1392 & -1972, *SRGN* ~-26427 [14], *SGK1* ~-1200 [12]. Overlapping regions identified in multiple studies were treated as single sites. |
| ***GEM, PLK2 & POMC*** | Primary endogenous target genes that are repressed by agonist bound GR [13,20]. At *POMC*, BRG1 is required for promoter recruitment of GR, the NGFI-B orphan receptor & the HDAC2 deacetylase, & is associated with reduced Pol II promoter clearance [20]. In U2OS cells expressing GR, *GEM* was observed to be repressed 1.3-fold by 6 hr dex treatment ([18], GEO accession # GSE 11205), and *GEM* and *PLK2/SNK* were repressed 4x and 3.5x by 2 hr dex treatment [19]. GR binding sites identified in prior ChIP studies were: *GEM* -264 to -378 & -1721 to -1822, *PLK2* -1191 to -1304 & -2663 to -2756 [13] and *POMC* ~-400 [20]. |
| ***MYC*** | Differentially regulated by ARID1A and ARID1B variant hSWI/SNF complexes [21,22,23,24,25]. In U20S cells expressing GR, *MYC* was found to be repressed 1.9-fold by 6 hr dex treatment [18] and by 2.4-fold by 2 hr dex treatment [19]. |
| ***CDK1, E2F1, CCNE1, CCNB2 & CCNA1*** | Cell cycle control genes which are unregulated by GR, but are regulated by the ARID1A and 1B hSWI/SNF complexes similarly to *MYC*. *CDK1* is also known as *CDC2*. “*CCN*”… denotes “cyclin.” |
| ***CDKN1A*** | *CDKN1A/p21* is a critical cell cycle regulator & cyclin/CDK inhibitor, transactivation of which may be central to hSWI/SNF’s function as a tumor suppressor [26,27,28,29]. In contrast to *MYC*, *CDKN1A* transcription is repressed by the ARID1A complex in cycling, rather than differentiated cells [24,25].  *CDKN1A* was induced 4 to 10 fold by GR and 4hr dex treatment in SW-13 cell derivatives [11], but was not detectably induced in U2OS microarray studies [18,19] |
| ***CCND1*** | Repression of *CCND1/cyclin D1* by hSWI/SNF appears to be a major reason why reintroduction of hSWI/SNF into cells that lack it causes cell cycle arrest [26,30,31,32]. |
| ***CSF1*** | Dramatically upregulated when cells lacking hSWI/SNF have hSWI/SNF function restored to them [33,34]. NF1 recruits hSWI/SNF, which results in the “disruption” of one promoter nucleosome [35]. |
| ***CD44*** | *CD44* encodes a cell adhesion and signaling molecule which is overexpressed in some aggressive and invasive breast and prostate cancers, and is highly dependent on hSWI/SNF for expression [33,34]. |
| ***GAPDH*** | *GAPDH* is not bound by or regulated by hSWI/SNF or GR [36,37], and is included as a negative control. |
| ***UGT1A6 & UGT1A8*** | Two drug metabolism genes with distinct promoters and first exons and shared last exons. They have not been reported to be regulated by GR. Regulation by hSWI/SNF is unknown. |

Blue: genes activated by GR and Dex. Pink: genes repressed by GR and Dex. Grey: cell cycle control genes that are not regulated by GR and Dex in U20S cells. For citations given in the table, see Additional Methods, in supporting text file S1.
