# Supplementary material for: Multiple Distinct Stimuli Increase Measured Nucleosome Occupancy around Human Promoters
Source: PLoS One. 2011 Aug 11;6(8):e23490. doi: 10.1371/journal.pone.0023490 (PMC3154950; doi:10.1371/journal.pone.0023490)
Supplement: Table S2 — Genomic locations and accession numbers for genes on the array. All coordinates are relative to the HG18 human genome build. TSS: transcription start site. (DOC) [file pone.0023490.s022.doc]

| **Table S2: Genomic locations & accession numbers for genes on the array** | | | | |
| --- | --- | --- | --- | --- |
| **Gene Name** | **Genbank Accession # (1^o^ transcript)** | **Range tiled on array (hg18 build)** | **TSS** | **strand** |
| CCNA1 | [NM_003914.3](http://www.ncbi.nlm.nih.gov/entrez/query.fcgi?cmd=Search&db=Nucleotide&term=NM_003914&doptcmdl=GenBank&tool=genome.ucsc.edu) | chr13:35884633-35922008 | 35,904,409 | + |
| CCNB2 | [NM_004701.2](http://www.ncbi.nlm.nih.gov/entrez/query.fcgi?cmd=Search&db=Nucleotide&term=NM_004701&doptcmdl=GenBank&tool=genome.ucsc.edu) | chr15:57164612-57211535 | 57,184,612 | + |
| CCND1 | [NM_053056.2](http://www.ncbi.nlm.nih.gov/entrez/query.fcgi?cmd=Search&db=Nucleotide&term=NM_053056&doptcmdl=GenBank&tool=genome.ucsc.edu) | chr11:69145054-69185422 | 69,165,054 | + |
| CCNE1 | [NM_001238.1](http://www.ncbi.nlm.nih.gov/entrez/query.fcgi?cmd=Search&db=Nucleotide&term=NM_001238&doptcmdl=GenBank&tool=genome.ucsc.edu) | chr19:34974741-35014058 | 34,994,741 | + |
| CD44 | [NM_000610.3](http://www.ncbi.nlm.nih.gov/entrez/query.fcgi?cmd=Search&db=Nucleotide&term=NM_000610&doptcmdl=GenBank&tool=genome.ucsc.edu) | chr11:35091993-35141993 | 35,116,993 | + |
| CDK1 | [NM_001786.4](http://www.ncbi.nlm.nih.gov/entrez/query.fcgi?cmd=Search&db=Nucleotide&term=NM_001786&doptcmdl=GenBank&tool=genome.ucsc.edu) | chr10:62188242-62230930 | 62,208,242 | + |
| CDKN1A1 | [NM_078467.1](http://www.ncbi.nlm.nih.gov/entrez/query.fcgi?cmd=Search&db=Nucleotide&term=NM_078467&doptcmdl=GenBank&tool=genome.ucsc.edu) | chr6:36734465-36770086 | 36,754,465 | + |
| CSF1 | [NM_000757.4](http://www.ncbi.nlm.nih.gov/entrez/query.fcgi?cmd=Search&db=Nucleotide&term=NM_000757&doptcmdl=GenBank&tool=genome.ucsc.edu) | chr1:110234980-110280877 | 110,254,980 | + |
| CYP3A4 | [NM_017460.3](http://www.ncbi.nlm.nih.gov/entrez/query.fcgi?cmd=Search&db=Nucleotide&term=NM_017460&doptcmdl=GenBank&tool=genome.ucsc.edu) | chr7:99185540-99239744 | 99,219,744 | - |
| E2F1 | [NM_005225.2](http://www.ncbi.nlm.nih.gov/entrez/query.fcgi?cmd=Search&db=Nucleotide&term=NM_005225&doptcmdl=GenBank&tool=genome.ucsc.edu) | chr20:31720150-31757854 | 31,737,871 | - |
| GAPDH | [NM_002046.3](http://www.ncbi.nlm.nih.gov/entrez/query.fcgi?cmd=Search&db=Nucleotide&term=NM_002046&doptcmdl=GenBank&tool=genome.ucsc.edu) | chr12:6493918-6524797 | 6,513,918 | + |
| GEM | [NM_181702.2](http://www.ncbi.nlm.nih.gov/entrez/query.fcgi?cmd=Search&db=Nucleotide&term=NM_181702&doptcmdl=GenBank&tool=genome.ucsc.edu) | chr8:95323663-95363733 | 95,343,733 | - |
| HSD11B2 | [NM_000196.3](http://www.ncbi.nlm.nih.gov/entrez/query.fcgi?cmd=Search&db=Nucleotide&term=NM_000196&doptcmdl=GenBank&tool=genome.ucsc.edu) | chr16:66002537-66035953 | 66,022,537 | + |
| MYC | [NM_002467.4](http://www.ncbi.nlm.nih.gov/entrez/query.fcgi?cmd=Search&db=Nucleotide&term=NM_002467&doptcmdl=GenBank&tool=genome.ucsc.edu) | chr8:128787498-128837853 | 128,817,498 | + |
| PCK1 | [NM_002591.3](http://www.ncbi.nlm.nih.gov/entrez/query.fcgi?cmd=Search&db=Nucleotide&term=NM_002591&doptcmdl=GenBank&tool=genome.ucsc.edu) | chr20:55549543-55581922 | 55,569,543 | + |
| PLK2 | [NM_006622.2](http://www.ncbi.nlm.nih.gov/entrez/query.fcgi?cmd=Search&db=Nucleotide&term=NM_006622&doptcmdl=GenBank&tool=genome.ucsc.edu) | chr5:57778569-57811670 | 57,791,670 | - |
| POMC | [NM_000939.2](http://www.ncbi.nlm.nih.gov/entrez/query.fcgi?cmd=Search&db=Nucleotide&term=NM_000939&doptcmdl=GenBank&tool=genome.ucsc.edu) | chr2:25230226-25265063 | 25,245,063 | - |
| SDPR | [NM_004657.5](http://www.ncbi.nlm.nih.gov/entrez/query.fcgi?cmd=Search&db=Nucleotide&term=NM_004657&doptcmdl=GenBank&tool=genome.ucsc.edu) | chr2:192400288-192444226 | 192,420,226 | - |
| SGK1 | [NM_005627.3](http://www.ncbi.nlm.nih.gov/entrez/query.fcgi?cmd=Search&db=Nucleotide&term=NM_005627&doptcmdl=GenBank&tool=genome.ucsc.edu) | chr6:134525082-134557695 | 134,537,727 | - |
| SLC19A2 | [NM_006996.2](http://www.ncbi.nlm.nih.gov/entrez/query.fcgi?cmd=Search&db=Nucleotide&term=NM_006996&doptcmdl=GenBank&tool=genome.ucsc.edu) | chr1:167692792-167741865 | 167,721,832 | - |
| SRGN | [NM_002727.2](http://www.ncbi.nlm.nih.gov/entrez/query.fcgi?cmd=Search&db=Nucleotide&term=NM_002727&doptcmdl=GenBank&tool=genome.ucsc.edu) | chr10:70497834-70541571 | 70,517,834 | + |
| TSC22D3 | [NM_004089.3](http://www.ncbi.nlm.nih.gov/entrez/query.fcgi?cmd=Search&db=Nucleotide&term=NM_004089&doptcmdl=GenBank&tool=genome.ucsc.edu) | chrX:106836113-106866947 | 106,846,947 | - |
| UGT1A6 | [NM_205862.1](http://www.ncbi.nlm.nih.gov/entrez/query.fcgi?cmd=Search&db=Nucleotide&term=NM_205862&doptcmdl=GenBank&tool=genome.ucsc.edu) | chr2:234245060-234353684 | 234,265,060 | + |
| UGT1A8 | [NM_019076.4](http://www.ncbi.nlm.nih.gov/entrez/query.fcgi?cmd=Search&db=Nucleotide&term=NM_019076&doptcmdl=GenBank&tool=genome.ucsc.edu) | chr2:234171030-234353684 | 234,191,030 | + |
| ZBTB16 | [NM_006006.4](http://www.ncbi.nlm.nih.gov/entrez/query.fcgi?cmd=Search&db=Nucleotide&term=NM_006006&doptcmdl=GenBank&tool=genome.ucsc.edu) | chr11:113410641-113460640 | 113,435,641 | + |

All coordinates are relative to the HG18 human genome build. TSS: transcription start site.
